# Genome assembly and population genomic analysis provide insights into the evolution of modern sweet corn

**DOI:** 10.1038/s41467-021-21380-4

**Published:** 2021-02-23

**Authors:** Ying Hu, Vincent Colantonio, Bárbara S. F. Müller, Kristen A. Leach, Adalena Nanni, Christina Finegan, Bo Wang, Matheus Baseggio, Carter J. Newton, Emily M. Juhl, Lillian Hislop, Juan M. Gonzalez, Esteban F. Rios, L. Curtis Hannah, Kelly Swarts, Michael A. Gore, Tracie A. Hennen-Bierwagen, Alan M. Myers, A. Mark Settles, William F. Tracy, Marcio F. R. Resende

**Affiliations:** 1 Horticultural Sciences Department, University of Florida, Gainesville, FL USA; 2 Department of Molecular Genetics and Microbiology, University of Florida, Gainesville, FL USA; 3 Cold Spring Harbor Laboratory, One Bungtown Road, Cold Spring Harbor, New York, NY USA; 4 Plant Breeding and Genetics Section, School of Integrative Plant Science, Cornell University, Ithaca, NY USA; 5 Roy J. Carver Department of Biochemistry, Biophysics, and Molecular Biology, Iowa State University, Ames, IA USA; 6 Department of Agronomy, College of Agricultural and Life Sciences, University of Wisconsin-Madison, Madison, WI USA; 7 Agronomy Department, University of Florida, Gainesville, FL USA; 8 Gregor Mendel Institute, Austrian Academy of Sciences, Vienna BioCenter, Vienna, Austria; 9 Present Address: Seneca Foods Corporation, LeSueur, MN USA; 10 Present Address: Applied Plant Sciences Graduate Program, University of Minnesota, St. Paul, MN USA; 11 Present Address: Bioengineering Branch, NASA Ames Research Center, MS 239-15, Moffett Field, CA USA

**Keywords:** Agricultural genetics, Evolutionary biology, Plant breeding

## Abstract

Sweet corn is one of the most important vegetables in the United States and Canada. Here, we present a de novo assembly of a sweet corn inbred line Ia453 with the mutated shrunken2-reference allele (Ia453-*sh2*). This mutation accumulates more sugar and is present in most commercial hybrids developed for the processing and fresh markets. The ten pseudochromosomes cover 92% of the total assembly and 99% of the estimated genome size, with a scaffold N50 of 222.2 Mb. This reference genome completely assembles the large structural variation that created the mutant *sh2-R* allele. Furthermore, comparative genomics analysis with six field corn genomes highlights differences in single-nucleotide polymorphisms, structural variations, and transposon composition. Phylogenetic analysis of 5,381 diverse maize and teosinte accessions reveals genetic relationships between sweet corn and other types of maize. Our results show evidence for a common origin in northern Mexico for modern sweet corn in the U.S. Finally, population genomic analysis identifies regions of the genome under selection and candidate genes associated with sweet corn traits, such as early flowering, endosperm composition, plant and tassel architecture, and kernel row number. Our study provides a high-quality reference-genome sequence to facilitate comparative genomics, functional studies, and genomic-assisted breeding for sweet corn.

## Abstract (web summary)

Sweet corn is one of the most important vegetables in North America and has undergone different selection pressures than non-sweet cultivars. Here, the authors report its genome assembly and reveal the evolutionary history of modern sweet corn through population genomic analyses.

## Introduction

Sweet corn (*Zea mays* L.) is grown all over the world and is one of the most important vegetables in the United States and Canada^1^. In the United States, sweet corn is a quintessential summer food, celebrated in local communities with festivals during harvest times. As a specialty crop, sweet corn has a farm gate value of $1.4 billion per year, divided into fresh (~74% of total value) and processing (canned and frozen corn) markets^2^. This starchy vegetable is a good source of dietary fiber, folate, niacin, essential amino acids, and lutein and zeaxanthin—two non-provitamin A carotenoids important in maintaining eye health and reducing the risk of age‐related macular degeneration^3^.

**Fig. 1:**
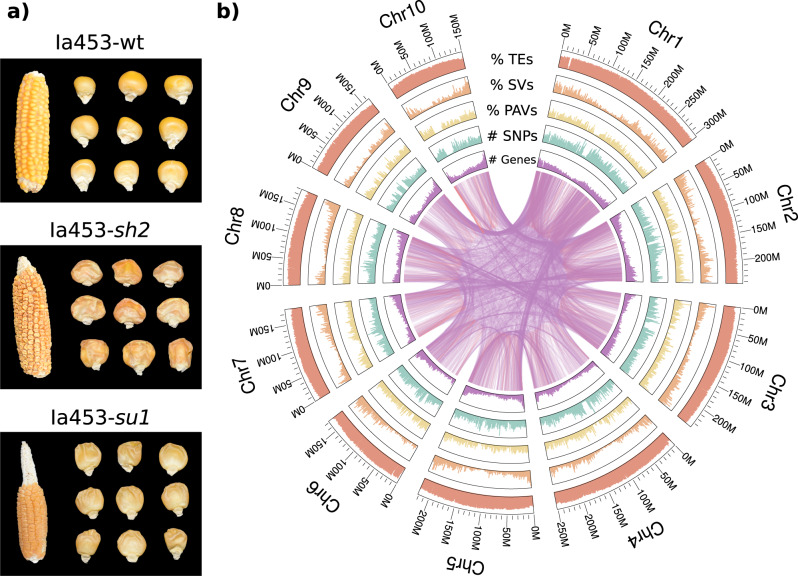
Appearance of sweet corn in Ia453 isolines and Ia453-*sh2* genomic features. **a** Visual ear appearance of sweet corn in Ia453 isolines, including wild-type (top), the *su1* mutation (bottom), and *sh2* mutation (center). **b** Ia453-*sh2* genomic features. Outer-to-inner tracks indicate the following: transposable-elements (TEs) (fraction of TEs per 1 Mb window); large structural variations (SVs) with length between 100 and 100,000 bps (fraction of SVs per 1 Mb window) relative to B73 v4; presence and absence variants (PAV) (fraction of PAVs per 1 Mb window) relative to B73 v4; single nucleotide polymorphisms (SNPs) (number of SNPs per 1 Mb window) relative to B73 v4; gene density (number of genes per 1 Mb window); and synteny with lines representing paralogous gene pairs between and within pseudochromosomes in Ia453-*sh2* (purple and orange lines).

Sweet corn is the result of mutations in genes involved in the starch biosynthesis pathway, which modify the carbohydrate composition by increasing sugar content in the endosperm while reducing starch content. Several genes in the starch biosynthetic pathway have been shown to increase sugar content in the endosperm when mutated^4–8^. Early commercial sweet corn hybrids increased sugar content by exploiting mutations in the *sugary1* (*su1*) gene, a starch debranching enzyme. Due to this defective enzyme, the endosperm of a mature kernel will appear wrinkled and translucent (Fig. 1a). In the 1970s and 1980s, sweet corn breeders initiated the development of hybrids with mutations in the *shrunken2* (*Sh2*) gene, which revolutionized the industry by improving eating quality and shelf life^9^. The *shrunken2* gene encodes the large subunit of ADP–glucose pyrophosphorylase (AGPase), the first committed enzyme of the starch biosynthesis pathway^10^. Homozygous *sh2* mutants accumulate more sugar, and the mature kernels are angular and shriveled due to reduced amounts of starch (Fig. 1a). Compared to homozygous *su1* mutants in which some sugars are converted to water soluble polysaccharides, homozygous *sh2* mutants have a higher sugar content at harvest, and this content has a slower rate of decline postharvest. Therefore, today, approximately 75% of the processing industry and nearly 100% of the fresh market industry utilizes hybrids containing the *sh2* mutation^11^.

In addition to the endosperm composition, commercial sweet corn hybrids are significantly different in phenotypic appearance from non-sweet maize hybrids, also known as field corn—one of the most widely produced cereals in the world. As a specialty vegetable, sweet corn plants have been selected for table quality traits such as kernel tenderness and color, and several esthetic traits that influence consumer acceptance, such as husk appearance, and silk and cob color. Furthermore, the plants are typically shorter, tend to flower earlier, and have a larger male inflorescence (tassel) than field corn^12^. Divergent selection between sweet and field corn predicts that a reference sweet corn genome will be considerably different than the field corn genomes previously assembled. To test this hypothesis, we sequenced a sweet corn inbred line, Ia453 with the *sh2-R* allele (Ia453-*sh2*). Ia453-*sh2* is an important public inbred line which contributed to the establishment of *sh2* sweet corn and is one of the parents of Illini Chief and Illini Xtra Sweet, two of the first commercial *sh2* sweet corn hybrids.

Here, we provide a high-quality reference genome of sweet corn through a combination of single-molecule real-time (SMRT) long-read sequencing, BioNano optical mapping, and Dovetail Hi–C mapping technologies. Ia453-*sh2* structural and genetic variations are identified through pairwise comparisons between Ia453-*sh2* and six field corn genomes. Phylogenetic analysis of 5381 maize and teosinte genotypes reveals genetic relationships among teosinte, landraces, modern sweet corn, and a diverse set of field corn lines. Finally, population genomic analysis identifies regions of the genome under selection and candidate genes associated with sweet corn traits, such as early flowering, higher sugar content, and tassel architecture. Our study provides an important resource to facilitate comparative genomics, functional studies, and genomic-assisted breeding for sweet corn.

## Results

### Genome sequencing and assembly

Four data sources were used to assemble the Ia453-*sh2* genome (Fig. 1b, Supplementary Fig. 1). First, 150.5 Gb (~70-fold coverage, 19.9 million reads) of PacBio single-molecule long reads were self-corrected and assembled with Canu^13^, generating 15,550 contigs with an N50 of 0.39 Mb. BioNano optical maps were generated to anchor the PacBio contigs into 29 super scaffolds and 8486 unscaffolded contigs with an N50 of 120.9 Mb. To further anchor and orient the super scaffolds and unscaffolded contigs into pseudochromosomes, Dovetail Hi–C mapping was used for scaffolding through a hierarchical clustering strategy^14^. The Hi–C assembly was polished with ntEdit^15^ using short-read Illumina data. The final assembly has a genome length of 2.29 Gb and contains 10 long super scaffolds, hereafter denoted as pseudochromosomes, with a total length of 2.11 Gb as well as 8440 unassigned contigs with a total length of 177.23 Mb. The pseudochromosomes covered 92% of the total assembly and 99% of the estimated genome size based on *k*-mer analysis (2.13 Gb), with a scaffold N50 of 222.2 Mb (Table 1, Supplementary Table 1). Flow cytometry analysis estimated that Ia453-*sh2* is 4.8% larger than B73 (Supplementary Table 2).

**Table 1:** The summary statistics of the sweet corn Ia453-*sh2* assembly.

Genomic feature	Ia453-*sh2*
Length of Ia453-*sh2* assembly (bp)	2,285,829,126
Length of 10 pseudochromosomes (bp)	2,108,596,175 (92.25%)
Maximum scaffold length (bp)	304,492,077
Scaffold N50 (bp)	222,201,399
Number of unassigned contigs	8440
Number of genes	38,384
Number of genes in ten pseudochromosomes	37,884 (98.69%)
Number of transcripts	72,765
Genes with RNAseq support	24,683
Total size of transposable elements (bp)	1,689,995,319

The quality and completeness of Ia453-*sh2* genome was evaluated through BUSCO and long-terminal repeat (LTR) assembly index (LAI) analysis^16,17^. BUSCO analysis showed that 94.6 % (1,363), 1.11 % (18), and 4.09 % (59) of the Plantae BUSCO genes are present in the assembled Ia453-*sh2* genome as complete, fragmented, and missing genes, respectively. Out of the 94.6% complete genes, 88.05% were single-copy genes and 6.59% were duplicated genes. These results are similar to what was obtained for field corn reference genomes such as B73 v4^18^, W22^19^, Mo17^20^, F7, EP1, and DK105 genomes^21^ (hereafter denoted as field corn genomes) (Supplementary Table 3). The LAI score presents the proportion of intact LTR sequences in the genome and can be used to evaluate the assembly continuity and completeness (Supplementary Fig. 2). The Ia453-*sh2* assembly resulted in a mean LAI score of 28.2 and was higher than the predominant field corn reference genome (B73 v4), indicating higher continuity and completeness of our assembly.

### Protein-coding gene annotation

The protein-coding genes of the Ia453-*sh2* genome were annotated by the MAKER‐P pipeline^22^ using RNA-seq and full-length cDNA data from seven different tissues of Ia453-*sh2* as well as transcripts and proteins from eight published genome annotations as evidence (Supplementary Fig. 3). The combination of gene ab initio prediction tools AUGUSTUS^23^ and FGENESH^24^ were trained using AUGUSTUS databases “maize5” and “monocots” for gene prediction. After removing putative transposons and low-confidence genes, 38,384 high-confidence protein-coding genes and 72,765 transcripts were identified. RNA-seq data supported 64% of the predicted genes. Nearly all (98.69%) of the predicted gene models are located on the 10 pseudochromosomes, with only 500 gene models (1.31%) located on unassigned contigs (Table 1).

### Transposon annotation

Transposable elements (TEs) were annotated in Ia453-*sh2* and the six field corn genomes (B73, Mo17, W22, EP1, F7, and DK105) with uniform methods to maintain consistency. Our pipeline identified a total of 2,647,709 TE elements in Ia453-*sh2* divided in 17 super-families. The TE elements were well distributed on the ten pseudochromosomes with a small increase in content near the centromeres (Fig. 1b, Supplementary Fig. 1, Supplementary Data 1). The TE annotation of Ia453-*sh2* covered 1.69 Gb, which represents 82.69% of the genome, including retrotransposon (69.85%) and DNA transposon (12.84%). The two most prevalent super-families were both long terminal repeat (LTR) retrotransposons, consisting of 843,793 (784 Mb total) Gypsy elements and 456,321 (420 Mb total) Copia elements. The 418,078 terminal inverted repeat (TIR) elements identified account for 140 Mb, while Helitrons were predicted to cover 104 Mb of the genome in 350,444 elements. Compared to all six field corn genomes, Ia453-*sh2* contains the lowest number of TEs and the lowest percentage of the genome length covered by TEs (Supplementary Data 1). However, the fraction of Ia453-*sh2* covered by LTR-Copia-like retrotransposon (20.58%), TIR Pif/Harbinger (0.56%), and MITE Tc1/Mariner (0.08%) represented the largest percentages compared to all six field corn genomes, with a relative increase in the percentage of each family ranging from 12 to 23% compared to the average content in the field corn genomes (Supplementary Data 1). These analyses illustrate the high level of divergence in TE families between maize lines, which results in structural and sequence variation of protein-coding genes and their regulatory sequences.

### Structural variation of *sh2-R* allele

**Fig. 2:**
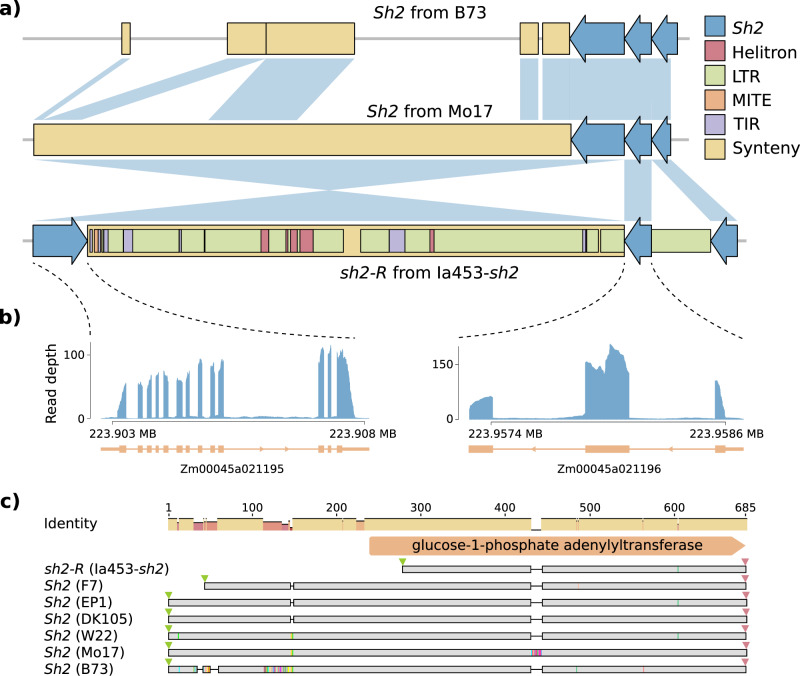
Structural variation of the *sh2*-*R* allele. **a** Scheme of the structural variations between *Sh2* in B73 and *Sh2* in Mo17 (the top pairwise comparison) as well as *Sh2* in Mo17 and *sh2-R* in Ia453-*sh2* (the bottom pairwise comparison). The *Sh2* in B73 and Mo17 is drawn the same size as the *sh2*-R allele in Ia453-*sh2* (blue arrows). Features within each allele are drawn to scale. The syntenic regions were drawn as yellow boxes and connected using blue wedges. The annonated TE elements are only shown in Ia453-*sh2* for simplicity. Two insertion sequences in the *sh2*-*R* allele contain a variety of predicted TE elements. **b** Two predicted gene models annotated in the *sh2-R* loci and their expression profile represented as the number of reads per Gb of total RNA-seq aligned reads. **c** Comparison of the predicted amino acid sequences of *sh2-R* (Zm00045a021195) from Ia453-*sh2* with *Sh2* from six field corn lines, using the Geneious multiple sequence alignment. Conserved amino acids are marked in gray, gaps are indicated as lines and differences are highlighted with different colors. The identity panel displays the identity across all amino acid sequences for every position. Small green and red triangles are indicating start and stop codons, respectively.

The *sh2-R* allele is a loss-of-function allele that conditions high sugar content at eating stage and propelled the modern sweet corn industry. At least two structural rearrangements occurred in *sh2-R* relative to the *Sh2* allele in field corn. In Ia453-*sh2*, the first half of the gene was separated by a 5.45 kb Copia LTR retrotransposon, which is intact, conserved, and young (insertion time estimated as zero due to conserved sequence) (Fig. 2a, Supplementary Table 4). The second half of the gene is inverted and separated from the first half by 49.44 kb of TE sequence, including one intact Copia LTR retrotransposon, one intact Gypsy LTR retrotransposon, and a variety of predicted TE events. This large TE sequence is present downstream of *Sh2* in Mo17 with 99.6% similarity but partially absent in B73 (Fig. 2a). This results supports the intra-chromosomal inversion proposed by Kramer et al.^25^, and here we show that this inversion happened in the middle of the gene (Fig. 2a). The insertion of the two intact LTRs in the 49.44 kb TE sequences was estimated to occur about 0.29 and 0.58 million years ago, but these insertions likely preceded the inversion (Supplementary Table 4). The inversion causes two separate gene models to be predicted in the *sh2-R* locus (Zm00045a021196 and Zm00045a021195) (Fig. 2b). Zm00045a021196 includes the fourth *Sh2* exon as well as two additional exons with gene expression support. Zm00045a021195 includes the last 13 exons of *Sh2*. Both genes are expressed in the endosperm, but Zm00045a021195 encodes an incomplete glucose-1-phosphate adenylyltransferase domain missing 36 amino acids in the N-terminus of the conserved domain (Fig. 2c).

### Global genomic comparison of Ia453-*sh2* to field corn genomes

A pairwise genomic comparison was performed between Ia453-*sh2* and six field corn genomes. One-to-one pairwise genome alignment resulted in an average syntenic match of 68.95%. The three flint (F7, EP1, and DK105) genomes had a higher percentage of syntenic matches (69.86%) compared to dent (B73, Mo17, and W22) genomes (68.04%) (Supplementary Table 5). In addition, in those one-to-one aligned regions, we identified an average of 10,493,202 SNPs between Ia453-*sh2* and the surveyed field corn genomes (Supplementary Table 5). The number of small insertions and deletions (indels <100 bp) in those one-to-one aligned regions averaged 806,274 and accounted for about 0.2% of the Ia453-*sh2* genome (Supplementary Table 5). Notably, there were fewer SNPs and small indels between DK105 and Ia453-*sh2* than other comparisons, suggesting a closer relationship between these two lines. The Ia453-*sh2* specific regions were also detected by comparing Ia453-*sh2* and other six field corn genomes using a sliding-window method. We identified between 56–61 Mb sequences that are specific in Ia453-*sh2* compared to B73, Mo17, W22, EP1, F7, and DK105, accounting for ~2.5% of the genome. An overlap of these unique regions with our TE annotation indicated that 68–69% of the specific sequences were found to overlap with TEs in all six comparisons (Supplementary Table 5). Large structural variations (SV) (100–100,000 bp) were detected with an average number of 23,664 compared against the field corn genomes, which encompassed, on average, a total length of 351.59 Mb (Supplementary Data 2). Repeat contraction or expansions made up the majority of SV sequences (88–90%) in all six comparisons (Supplementary Data 2).

### Pan-gene and gene structural variation analysis

The distribution of orthologous gene families in Ia453-*sh2* and all six field corn genomes was defined using OrthoMCL^26^. We identified an average of 22,322 core genes belonging to gene families that were shared by all seven corn genomes, 16,667 dispensable genes missing orthologous counterparts in at least one of the tested genomes, and 5545 singleton genes assigned to families which were unique to only one line (Supplementary Fig. 4). Out of the set of 22,322 core genes, we identified a set 7864 highly conserved protein coding genes using pairwise comparative analysis of the sweet corn genome with each of the six field corn genomes. These genes were functionally enriched in conserved biological processes and molecular functions, such as pre-mRNA 5′-splice site binding (GO:0003843, *p* value = 1.1e − 4) or photoreactive repair (GO:0000719, *p* value = 8.4e − 4) (Supplementary Data 3). This pairwise comparative analysis also resulted in a set of Ia453-*sh2* specific genes that could not be aligned to other field corn genomes or that were aligned with very low coverage and identity (Supplementary Fig. 5). We identified, on average, 148 genes specific to Ia453-*sh2* when individually compared with each field corn genome (Table 2), with six of these genes found uniquely in the Ia453-*sh2* genome (Supplementary Table 6). The same analysis in B73 resulted in similar number of genes unique to B73 (Table 2).

**Table 2:** Summary of the Ia453-*sh2*- and B73-specific genes compared to other six corn genomes.

Genes specific to	Number of genes calculated from the pairwise comparison	Ia453-*sh2*	B73	Mo17	W22	DK105	EP1	F7
Ia453*-sh2*	Genes in Ia453-*sh2-*specific regions	–	364	425	455	387	357	403
	Genes from Ia453-*sh2* with low identity or absent for the pairwise comparison	–	574	665	661	594	562	614
	Overlap	–	127	184	186	132	124	137
B73	Genes in B73-specific regions	340	–	423	455	383	369	420
	Genes from B73 with low identity or absent for the pairwise comparison	827	–	941	1102	926	911	975
	Overlap	122	–	161	186	115	115	143

Among the six Ia453-*sh2-*specific genes, Zm00045a011525 encodes an acetylating enzyme methylmalonate–semialdehyde dehydrogenase, a gene previously found to affect seed storage reserve and germination rate^27^. Zm00045a021614 encodes an F-box, LRR, and FBD domain containing protein, which plays a role in plant immune responses through involving in hormone pathways or functioning in plant–pathogen interactions^28,29^. Zm00045a046064 encodes a PAE1 proteasome subunit alpha type-5, which is involved in protein and RNA degradation, and is associated with the plant response to the majority of stresses^30^. Zm00045a030178 encodes a sphingosine kinase2, which is involved in the production of sphingolipid metabolites and abscisic acid (ABA) signaling that mediates stomatal closure, inhibition of seed germination, and root elongation^31^. The other two genes (Zm00045a028561 and Zm00045a030528) are predicted to encode proteins with unknown functions. None of those six genes had premature stop codons and all of them had proper annotations that agree with aligned ESTs from other species, and protein homology data based on annotation edit distance (AED) score (AED: 0.11–0.42). Furthermore, the MAKER mRNA quality index (QI), another quality metric to dissect the gene annotation, showed that two genes (Zm00045a030178 and Zm00045a046064) had support from splice-sites confirmation (QI2 = 0.85 and 0.50, respectively). In addition, these two genes were expressed in kernel, leaf, stem, silk, husk, ear, and pollen tissues, while the other four genes were not detected to be expressed in the same tissues (Supplementary Fig. 6). These results were further supported by the analysis of publicly available 3′-RNA-seq^32^ from five sweet corn (P39, Il14H, Il677a, Il101T, and IA2132) and five field corn inbred lines (B73, Mo17, W22, EP1, and F7) where both genes were only expressed in sweet corn (Supplementary Fig. 6). Seed germination and response to biotic stress are traditionally important traits in sweet corn breeding, which could explain the presence of these two genes with detected gene expression. Further studies are required to validate their function and test if they are present in a broad sweet corn germplasm.

### Phylogenetic analysis traces the evolutionary origins of sweet corn

**Fig. 3:**
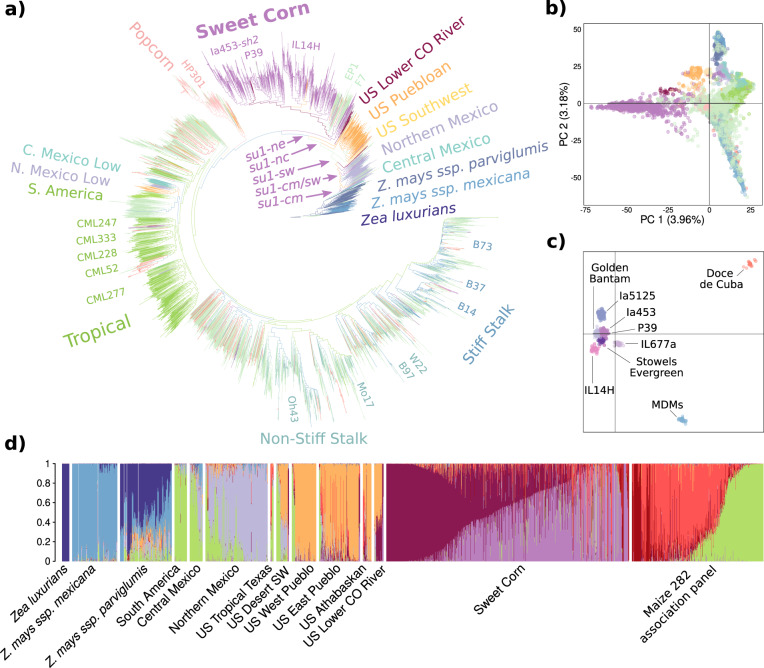
Phylogenetic relationships and population structures of diverse teosinte and maize accessions. **a** Phylogenetic tree of teosinte, landraces, sweet corn, and field corn genotypes. **b** Principal component analysis color-coded as in the phylogenetic tree. **c** Discriminant analysis of principal components (DAPC) plot with only the sweet corn lines using different colors, highlighting an important line that is representative of each cluster—“MDMs” label represent a group of sweet corn lines bred for Maize Dwarf Mosaic Virus. **d** Bayesian clustering of the population using STRUCTURE with *K* = 9. Sweet corn and Maize 282 association panel were sorted based on one of their prevalent populations. The other groups were sorted based on the altitude where they were grown according to Swarts et al.^57^. The colors used do not represent the same grouping as panels (**a**) and (**b**).

We evaluated genotypes of diverse maize accessions using a genotyping-by-sequencing build to help understand how sweet corn is related to other types of maize. The panel included teosinte, landraces, and field corn inbred lines in addition to 822 sweet corn lines. Leveraging RaXML-NG, we produced a maximum likelihood-based phylogenetic tree comprising 5381 diverse maize accessions (Fig. 3a). The tree was rooted with the teosinte *Zea luxurians* [Durieu and Asch.]. As expected, we found other teosintes including *Z. mays ssp. mexicana* [Schrad.], *Z. mays ssp. parviglumis* [Iltis and Doebley], and *Z. luxurians* to be sister clades to cultivated maize. Maize accessions from Central Mexico, Northern Mexico, US Southwest, and US Puebloan were found to subsequently diverge. The sweet corn accessions were primarily found in their own clade sister to the remaining maize populations. This is likely due to breeding of sweet corn specific traits, and the large contribution of a few sweet corn genotypes, such as Golden Bantam and Stowell’s Evergreen, to modern commercial varieties. Flint corn varieties including F7 and DK105 were found to be grouped with the sweet corn clade, supporting the hypothesis that modern sweet corn varieties share the origins or are primarily derived from Northern Flints^33^, a race of corn grown in the past in eastern North America. Clustering of the samples using STRUCTURE and discriminant analysis of principal components (DAPC) also showed the separation of sweet corn from the rest and further divided the sweet corn clade into different groups (Fig. 3c, d, Supplementary Fig. 7). STRUCTURE analysis with different group numbers (*k* = 4 to 16) was performed (Fig. 3d, Supplementary Fig. 7). The optimum number based on Evanno’s criteria was estimated as nine with well-defined groups for *Z. luxurians*, *Z. mexicana*, and landraces from South America, Northern Mexico, and US Puebloan. The marker set used was not able to allocate *Zea parviglumis* into its own group, presumably due to missing data or undersampling of genetic variability. The STRUCTURE cluster that contained the majority of sweet corn lines is represented with high membership probability (>0.99) by the variety Golden Bantam and inbred line P39, two important genotypes in the history of sweet corn breeding. A second large STRUCTURE cluster traces back with high membership probability to IL677a, a *su1* line which is also the source of the mutant gene *sugary enhancer* (*se*)^34^, a third mutant gene used in sweet corn breeding programs and recently cloned^35^. A DAPC calculated using only the sweet corn genotypes supported a cluster containing IL677a and further identified groups that contained additional lines known to have contributed to modern sweet corn breeding, such as Stowell’s Evergreen and Ia5125^4–8^ (Fig. 3c).

From an evolutionary perspective, sweet corn is known to have arisen multiple times as evidenced by the presence of multiple independent *su1* alleles^33^. We identified in the phylogenetic tree the samples containing the alleles identified by Tracy et al.^33^ to infer further the evolutionary history of *su1* genotypes (Fig. 3a). We found the Maiz Dulce variety Guanajuato, containing the *su1-cm* allele with a 1.3 kbp TE inserted in exon 1, to be grouped with the central Mexico landraces. The Ducillo de Noroeste variety from Sonora containing the *su1-sw* (N561S) allele was found to be clustered with the northern Mexico landraces. The southwestern 12 Row varieties, which included Tawa’ktci, Moencopi, and Hotevilla, were found to be clustered with Puebloan landraces from the southwestern United States. These landraces also contain the *su1-sw* allele suggesting a migration from northern Mexico to southwestern United States. The Nueta varieties derived from the Great Plains Flints and Flours containing the *su1-nc* (R504C) allele were found to be diverging near the flint varieties at the base of the sweet corn clade. Finally, the *sugary1-reference* 36 allele (also known as *su1-ne*—W578R) was placed in the group that contained the majority of sweet corn lines, as expected given that most modern sweet corn lines contain the *su1-ne* allele^33^.

**Fig. 4:**
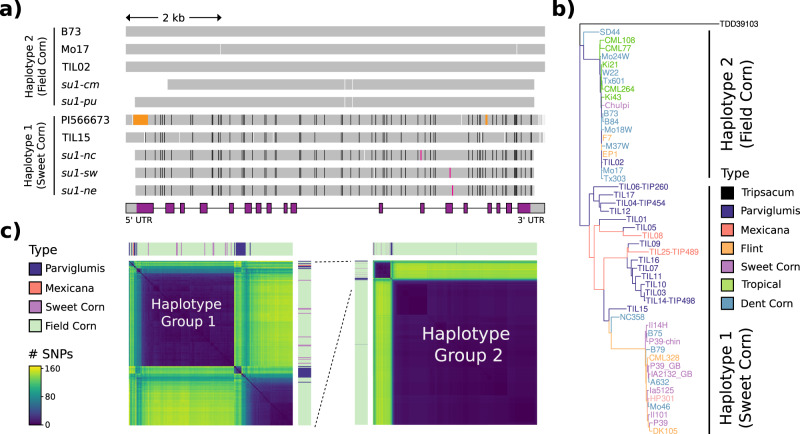
Two different haplotypes of the *su1* locus. **a** Haplotypes at the *su1* locus. Colored-coded vertical lines represent SNPs or deletions of 5 bp or less relative to the B73 reference sequence. Black lines denote SNPs present in *su1-ne*, white lines indicate SNPs in other alleles not shared with *su1-ne*, magenta lines indicate causative agents of the *su1-*defect, and orange blocks indicate deletions in PI566673^115^ (*Zea mays ssp. mexicana*) relative to B73. Every variation from B73 is indicated. In the gene model black lines indicate introns, gray bars indicate untranslated region exons, and purple bars indicate coding sequence exons. The figure is drawn to scale except for a few SNP locations that have been slightly adjusted to allow visual resolution. The sequences of regions not covered by gray bars were not determined. **b** Neighbor-joining *su1* tree rooted in *Tripsacum dactyloides*, containing teosinte samples and representatives of the two dominant *su1* haplotype groups. **c** Heatmap of the distance matrix calculated from the 165 SNPs within the full length *sugary1* gene. The left part of the panel represents a blowup of the full heatmap, presented on the right corner of the same panel. Haplotype group 1 is referred in the text as the sweet corn haplotype while haplotype 2 is the field corn haplotype.

We further explored the *su1* locus and identified the presence of two distinct groups of sequences for *su1*, hereafter denoted as major haplotype groups. While the five known alleles have independent origin^33^, as evidenced by distinct causative mutations, three of such *su1* alleles (*su1-nc, su1-sw, su1-ne*) are contained in one of the major haplotype group (referred as the “sweet corn” haplotype) that includes 165 SNPs relative to B73 (Fig. 4). These three alleles were the ones involved in the origins of sweet corn in North America. The other two *su1* sweet corn alleles, *su1-pu* in the Andean landrace “Chullpi”^37^ and *su1-cm* in the Maiz Dulce line from central Mexico, exhibit very few SNPs relative to B73 and thus contain the “field corn” haplotype of the *su1* locus (Fig. 4a). Extending this analysis to publicly available genomic sequences revealed distinct phylogenetic separation of the two haplotype groups (Fig. 4b). A k-means clustering of the B73 *sugary1* sequence updated with HapMap 3 SNPs^38^ shows that 114 lines have the “sweet corn” haplotype and 1065 have the “field corn” haplotype (Fig. 4c). The former group includes, in addition to most sweet corns, genotypes from teosinte (both *Z. parviglumis* and *Z. mexicana*), popcorn, flint lines, and a minority of field corn lines, whereas most field corn lines are represented by the second haplotype group (Fig. 4b,c). The *Z. parviglumis* genotype TIL02 was the only teosinte line assigned to the field corn haplotype group (Fig. 4a, b). Nucleotide diversity among corn samples was low in general, but particularly lower in Haplotype 2 (Π_HAP1_ = 0.00092; Π_HAP2_ = 0.00024; 11,598 bp), suggesting evidence of selection in this locus. Out of the landraces sequenced in HapMap3, the “sweet corn” haplotype is also present in Assiniboine (BKN014), Longfellow Flint (BKN016), Tabloncillo (BKN035), Poropo (BKN010), and Reventador (BKN022) (Supplementary Fig. 8). Interestingly, Reventador, a popcorn landrace from Mexico has been hypothesized to be one of the parents of the sweet corn landrace Ducillo del Noroeste and is also thought to be the ancestor of the Tabloncillo race^39^. Altogether, these results suggest a common origin in northern Mexico for modern sweet corn in the United States. prior to the natural creation of the three different *su1* alleles.

### Population genomic analyses reveal sweet corn specific selective sweeps

In addition to having low starch content, sweet corn varieties are morphologically and physiologically distinct from field corn. They are shorter, have larger tassels, flower earlier, and have droopier leaves. Sweet corn plants also tend to have more tillers, and a major QTL, *tin1*, associated with tillering growth habit of the sweet corn was recently reported^40^. In addition, sweet corn has been selected upon by breeders for different traits, including processor and consumer-oriented traits such as shape, size, flavor, and kernel row number. Population genomic analyses were used to scan the genome for signatures of selection with the hypothesis that these may contribute to characteristic sweet corn phenotypes. We calculated fixation index (*F*_ST_), Tajima’s *D* in 10 kb windows, and cross-population composite likelihood ratio (XP-CLR) as metrics of population differentiation between sweet corn populations (*su1*-type, *sh2-*type, and all sweet corn) and the maize 282 association panel.

**Fig. 5:**
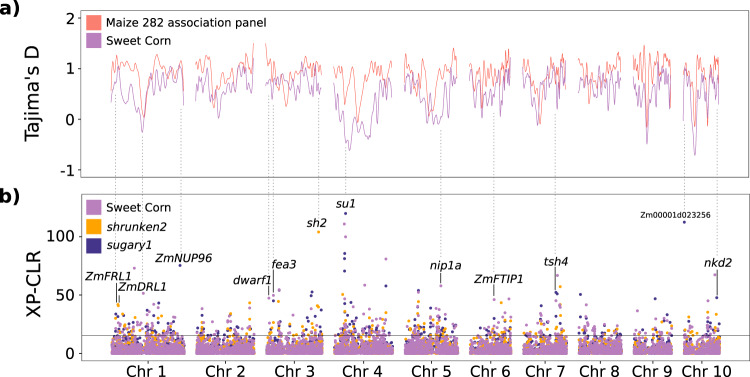
Genome-wide distribution of sweet corn specific selective sweeps. **a** Tajima’s *D* calculated on the sweet corn population and the maize 282 association panel (excluding the sweet corn lines within the maize 282 association panel). **b** XP-CLR estimated by comparing the maize 282 association panel against the sweet corn population (purple), the *shrunken2* subpopulation (orange), and the *sugary1* subpopulation (dark blue).

The *F*_ST_ between the sweet corn population and the maize 282 association panel (excluding the sweet corn lines in it) was 0.12, indicating a moderate level of differentiation. As expected, based on the Tajima’s *D* results, the regions around the *su1* and *sh2* loci show evidence of selection in sweet corn populations but not in maize 282 association panel varieties (Fig. 5a). In addition, distinct regions were found to have undergone selective sweeps on chromosomes 1, 4, and 5. Although the Tajima’s *D* at these loci suggest selection is at play, determining the causal genes can be difficult due to the size of the loci.

To detect selective sweeps, XP-CLR was calculated for 50 kb windows across the genome between the maize 282 association panel and the sweet corn population. The sweet corn population was also further sub divided into *su1* and *sh2* populations and compared against the maize 282 association panel. Using this approach, we identified windows with high XP-CLR peaks that overlapped with deviations in Tajima’s D. These windows included candidate genes known to be involved in traits that are characteristic of sweet corn, such as plant height, leaf angle, tassel and ear architecture, early flowering, endosperm composition, and tillering (Fig. 5). Specifically, we found regions containing the genes *drooping leaf1* (*ZmDRL1*; Zm00001d028216), *frigida-like protein1* (*ZmFRL1*; Zm00001d028173), *nuclear pore complex protein 96* (*ZmNUP96*; Zm00001d031680), *dwarf plant1 (dwarf1; Zm00001d039634), fasciated ears3* (*fea3*; Zm00001d040130)*, NOD26-like membrane intrinsic protein1* (*nip1a*; Zm00001d016237), *FT-interacting protein1* (*ZmFTIP1*; Zm00001d036804), *tassel sheath4* (*tsh4*; Zm00001d020941), and *naked endosperm2* (*nkd2*; Zm00001d026113) in addition to *sugary1* and *shrunken2*. The *drooping leaf1* encodes a CRC-like transcription factor and is associated with canopy structure and leaf angle^41^. In *Arabidopsis*, *FRIGIDA-LIKE PROTEIN1* (*FRL1*) is required for *FRIGIDA*-mediated upregulation of the *FLOWERING LOCUS C (FLC)*. The *frl1* mutant in *Arabidopsis* suppresses *FRIGIDA*-mediated late flowering and up-regulation of *FLC*^42^. *Dwarf1* locus encodes a gibberellin 3-oxidase, and the mutant leads to short stature plants^43^. Another XP-CLR window contains the *fasciated ears3* gene, a leucine-rich-repeat receptor, and variation in this locus can impact the thickness of tassels and kernel row number^44^. The presence of tillers in sweet corn plants, and large tassel architecture could in part be controlled by the candidate genes *nip1a*^45^, and *tassel sheath4*^46^, respectively. Two candidates for early flowering were also found, *FT-interacting protein 1*^47^ and *nuclear pore complex protein 96*^48^. A peak on chromosome 10 overlaps with candidate gene Zm00001d023256, predicted to encode an ADP glucose pyrophosphorylase small subunit. Finally, one of the regions under selection includes the candidate gene *naked endosperm 2*. The mutant for this locus produces multiple aleurone outer cell layers, affects starch content and composition as well as impacting greater than 6% of the transcriptome in these endosperm cell types^49,50^.

## Discussion

Neolithic people domesticated maize from teosinte about 9000 years before present (yr B.P.), most likely in southwestern Mexico and then maize was carried by early agriculturalists throughout the Americas. Archeological evidence indicates that maize was being cultivated in what is now the southwestern United States about 4000 yr B.P^51–54^. By 1000 yr B.P., maize was already widely cultivated and a staple in the diet of the Eastern Woodlands people^55^. It was then rapidly adopted by many the people of eastern North America^56,57^. During this time, sweet corn was grown and maintained by indigenous peoples^39,58–61^, with modern sweet corn found to be related to Puebloan landraces grown by Native Americans in what is now the southwestern United States. Mutant *su1* alleles have been fixed and deliberately cultivated at least three different times in North America. The presence of three of the alleles in a conserved haplotype suggests a potential common origin. The *su1* haplotype also seems to generally coincide with the origin of ‘Northern Flints’ in North America. It is unclear why this haplotype would have been maintained, although low recombination rates in this genetic background could explain this observation, which is supported by a large region with very low genetic diversity surrounding the *su1* locus on chromosome 4 (Fig. 5).

The *shrunken2* mutation is thought to be more recent, with records dating back to a maize stock from professor E.B. Mains created in 1943^62^. Previous work studying the *sh2* gene documented the presence of a large insertion containing a Helitron and a complex rearrangement that caused the phenotype^25^. Our sweet corn genome provides a complete and consensus *sh2-R* allele sequence, which highlights the role and importance of structural rearrangements as a mechanism to create genetic diversity in maize.

Sweet corn is an important starchy vegetable with specific breeding targets, which have created bottlenecked populations that are very distinct from field corn. As an example, we see little overlap in our results with genomic regions under selection in modern field corn, recently presented and discussed in Wang et al.^63^. The generation of specialized genomic resources for sweet corn can not only provide support for future marker-based breeding decisions but also contribute towards the characterization of genes potentially not variable in field corn. Advances in next-generation sequencing and physical mapping technologies have reduced costs to enable routine sequencing of maize genomes by combining multiple technologies. Here, we present a highly contiguous genome assembly for a sweet corn inbred line, Ia453-*sh2*, to complement other field corn genome assemblies and provide a resource for comparative genomics available for the sweet corn community. Furthermore, population genetic analysis provided a list of candidate regions that have been differentially selected in sweet corn and field corn. These regions reflect the differential breeding choices and breeding targets that shaped modern sweet corn.

## Methods

### Plant material

The sweet corn (*Z. mays*) inbred line Ia453 with *sh2*-R allele (Ia453*-sh2*) was sequenced. The maize plants utilized for Illumina sequencing were grown in the greenhouse complex at the University of Florida (Gainesville, FL) in November of 2018. Young leaves from 3-week-old plants were harvested and frozen in liquid nitrogen. Genomic DNA was extracted using a modified CTAB method^64^ with an RNase treatment carried out for 30 min. DNA from 1-week old etiolated seedings from the same seed source was extracted utilizing the same method for PacBio sequencing.

### PacBio and Illumina data generation

Large insert (20 kb) SMRTbell libraries were prepared and sequenced by the ICBR at the University of Florida using a PacBio SEQUEL system according to the recommended protocol (P/N 100-286-000 Version 10 January 2018) with a few modifications. Briefly, high-integrity genomic DNA was further cleaned using the MoBio PowerClean DNA Cleanup Kit (# 12877-50) kit. DNA was concentrated using AMPure beads (1:1 bead:sample ratio) and used for the subsequent SMRTbell library construction steps. The library construction steps included: ExoVII treatment, DNA Damage Repair, End Repair, Blunt-end ligation of SMRT bell adapters, and ExoIII/ExoVII treatment. The final library was size-selected in the SageELF^TM^ instrument (Cat# ELD 7510), using 0.75% agarose gel cassettes and the 1–18 kb v2 cassette definition program. The desired SageELF fractions were cleaned using AMPure magnetic beads (0.6:1.0 beads to sample ratio) and eluted in 15 µL of 10 nM Tris HCl, pH 8.0. Between 6 and 8 pM of library was loaded onto the PacBio SEQUEL sample plate for sequencing, using diffusion loading and 20 h movies (sequencing chemistry v3.0, SMRT Link 7.0). DNA extracted from the same sample was used to build standard 300-bp Illumina libraries. All Illumina libraries were prepared and sequenced with 150 bp paired-end reads on an Illumina Hiseq 2500 system at GENEWIZ Inc. (South Plainfield, NJ).

### PacBio long-read de novo assembly

Around 19.9 million PacBio SMRT SMRT subreads were error-corrected and assembled using Canu v1.8^13^. For the full data set, only reads longer than 5 kb were corrected using the parameter: minReadLength = 5000. By default, Canu only selects the longest 40× for correction. In order to get more corrected reads, the following parameter corOutCoverage = 60 was used and resulted in 44.54× corrected sequence. The read trimming and unitig construction were run with the default parameters. To further improve the accuracy of the reference assembly, arrow (https://github.com/PacificBiosciences/GenomicConsensus/) was used to correct the sequencing errors with default parameters.

### BioNano mapping construction and hybrid assembly

Ultra-high molecular weight DNA was isolated from leaf tissues using the BioNano Prep^™^ Plant Tissue DNA Isolation Kit. The DNA was then labeled using the BioNano Direct Label and Stain method according to a protocol developed by Bionano Genomics^65^. The BioNano Saphyr system was used to stretch, separate and image the labeled DNA molecules. The resulting BNX file with the raw digitized molecules was filtered to include only molecules longer than 250 kb with at least nine fluorescent labels. The filtered molecule data set had a molecule N50 of 383 Mb and was de novo assembled using the BioNano Solve 3.2 software. Maps were recursively refined and extended to construct the consensus maps. The total length of the final genome maps was 2.15 Gb, with a map N50 of 119.5 Mb and contained 69 BioNano genome maps. The PacBio contigs were in silico digested into consensus physical maps (CMAPs) and were compared with the genome maps for hybrid scaffolding using the BioNano Solve software. The scaffolding was visualized and curated with the BioNano Access software. When conflicts occurred, the contigs were edited in accordance of alignment between PacBio contigs and the BioNano genome map. A total of 6890 PacBio contigs were linked into 29 super-scaffolds with an N50 of 120.95 Mb and a total length of 2.14 Gb. There were 8660 unscaffolded PacBio contigs. The total length of the super-scaffolds and unscaffolded PacBio contigs was 2.32 Gb with an N50 of 120.95 Mb.

There were 4734 overlaps between the scaffolds from the hybrid assembly with BioNano. When two PacBio contigs are found to share sequences in their extremes, the software will output 13-N gaps between two contigs. Flanking 100 kb sequences around the 13-N gaps were extracted and merged by Minimus2 when they overlapped^66^. A total of 4316 scaffolds were successfully merged. A python-based command line tool reform (https://github.com/gencorefacility/reform) was used to put back the merged sequences into the hybrid assembly.

### Hi–C library preparation and sequencing

A Hi–C library (Dovetail Genomics LLC, Santa Cruz, CA) was generated using the *DpnII* restriction endonuclease (GATC). Briefly, this entailed reconstituting chromatin using purified histones and chromatin assembly factors, followed by cross-linking the chromatin using formaldehyde. DNA was then digested using *DpnII* restriction enzymes, 5′ overhangs filled in with biotinylated nucleotides and free blunt ends were ligated. After ligation, cross-links are reversed and the DNA was purified from protein. The DNA was then sheared to a mean fragment size of about 350 bp, and biotin-containing fragments were isolated using streptavidin beads. The resulting DNA fragments were taken through a standard Illumina library preparation and sequenced on HiSeq X-Ten (2 × 150 bp paired-end reads) to provide ~56× physical genome coverage.

### Pseudomolecule construction with HiRise

The Dovetail Hi–C library were used to scaffold the 29 super-scaffolds and 8660 unscaffoled PacBio contigs through Dovetail’s HiRise pipepline. Dovetail Hi–C library sequences were aligned back to the input de novo assembly using a modified version of SNAP (http://snap.cs.berkeley.edu/). The read pairs that mapped uniquely on the assembly were used to generate a likelihood model to estimate the genomic distance between read pairs, identify and break the putative misjoins, score the prospective joins and make joins above a selected threshold. There were no breaks and 12 joins made by HiRise. Dovetail HiRise assembly contains 10 chromosomes and 8440 unscaffolded contigs (Supplementary Table 7).

To further polish the pseudomolecules, a ~23× coverage of paired-end Illumina whole genome sequencing library was generated for sequencing polishing using ntEdit^15^. The ntHits was first ran with parameters “-k 25 -c 2” to build a Bloom filter, which is read by ntEdit to polish the assembly with default parameters. A total of 832,323 changes were corrected, including 31.29% SNPs and 68.7% small indels (2–25 bps).

### Genome size estimation

The genome size was estimated using k-mer histograms computed from the error-corrected PacBio reads using the program jellyfish v.2.3.0^67^, with word sizes (*k*) of 25. Furthermore, four genotypes were used to estimate genome size using flow cytometry. The B73 genotype was used as the reference standard and we estimated the genome size for Ia453-*sh2*, F7 and W22. Flow cytometry measurements were made on five biological replicates per genotype, and the genome size estimated as a ratio relative to B73 was reported as the average across the five replicates (Supplementary Table 2). Sample preparation: five seeds per genotype were imbibed for 48 h in full darkness, and the embryo was dissected and used for the analysis following the methodology described by Rios et al.^68^. Specifically, the embryo was mixed with 500 μl of extraction buffer (CyStain PI absolute P, Partec GmbH, Münster) and chopped using a sharp razor blade for 30 s in a petri dish kept on ice. The excised tissue was then incubated on ice for another 30 s. Samples were then filtered using Partec 50 μm CellTrics (Partec GmbH, Münster), and stained using 2 ml of staining solution containing propidium iodide and RNase (CyStain PI absolute P, Partec GmbH, Münster). Samples were incubated on ice for at least 30 min and analyzed immediately with the BD Accuri C6 Flow Cytometer with laser illumination at 488 nm and a 610/20 nm filter with the FL2 detector (Accuri Cytometers, Ann Arbor, MI) at the University of Florida Interdisciplinary Center for Biotechnology Research, Gainesville, FL. The gating strategy is provided in the Supplementary Fig. 9. For each sample at least 5000 nuclei were counted and analyzed using the BD Accuri CFlow software (Version 1.0.264; BD Biosciences, CA, USA), and only samples whose G1 peak had a coefficient of variation (CV FL2-A) smaller than 10% were considered for analysis. The amount of DNA contained within diploid nuclei (2C-DNA content) for Ia453-*sh2*, F7 and W22 was calculated based on the fluorescence intensity recorded for their G1 peaks and compared to the G1 peak of the reference standard (B73).

### Assembly evaluation

The genome completeness from contig to chromosome-level assembly was assessed using the benchmarking universal single-copy orthologs (BUSCO) v3.02^17^. The final assembly was tested against the Plantae BUSCO “Embryophyta_odb9” database, which contained 1440 protein sequences and orthogroup annotations for major clades. This result was compared with that of B73, Mo17, W22, EP1, F7, and DK105 genomes. The assembly of line PH207 was not included due to lower assembly quality. The LAI—a method to evaluate genome assembly completeness based on the quality of the assembly of repeat sequences—was also run for all the genomes above using the LTR_retriever pipeline^16^.

### PacBio Iso-seq library preparation, sequencing, and assembly

Total RNA was extracted from leaf, stem, silk, husk, ear and pollen tissues using Trizol and RNeasy MinElute (Qiagen) RNA clean up kit, with an Dnase I treatment of 20 min^69^. The RNA integrity was assessed with a Bioanalyzer prior to the construction of the Iso-Seq library. The Iso-Seq libraries were prepared and sequenced by Interdisciplinary Center for Biotechnology Research (ICBR) at the University of Florida using a PacBio Sequel system. PacBio Iso-Seq data were analyzed by running the IsoSeq3 v3.1 in PacBio SMART Analysis v7.0 (https://github.com/PacificBiosciences/IsoSeq3) to generate high-quality, full-length transcript sequences.

### RNA-seq and transcriptome assembly

RNA-seq data was also generated for endosperm sampled 14 days after pollination. The total RNA was extracted using RNeasy MinElute (Qiagen) following the manufacturer’s recommended protocol. The total RNA was processed using the TruSeq RNA Sample Preparation kit followed by sequencing on the Illumina HiSeq 2500 platform. The software Trimmomatic v0.36 was used to trim adapter sequences of RNA sequencing reads^70^. The paired-end reads were merged using PEAR v0.9.6^71^, which were used for following transcriptome assembly. The de novo transcriptome assembly was performed using Trinity v2.8.4 with default parameters^72^. The genome-guided transcriptome assembly was performed with HISAT2 v2.1.0^73,74^ and StringTie v1.3.4^75^. The genome index was built using HISAT2-build and the clean transcriptome reads were mapped to the sweet corn genome using HISAT2. The genome-guided transcriptome assembly was performed using StringTie. The resulting StringTie and Trinity assemblies were supplied to PASA v2.2.0^76^ in order to build comprehensive transcriptome database.

### Protein-coding gene annotation

MAKER-P v2.31.10^22^ was used to annotate genes in the Ia453*-sh2* genome with the evidence data from the annotation of the B73v4 genome and some additional evidence as outlined below. RepeatMasker was used to mask low complexity genomic sequence using exemplar transposon sequences^77^. The annotated proteins from *Sorghum bicolor*, *Oryza sativa*, *Setaria italica*, *Brachypodium distachyon*, and *Arabidopsis thaliana*, downloaded from Gramene.org release 48^78^, were used for protein evidence. A set of 69,163 publicly available full-length cDNAs and 2,019,896 publicly available ESTs deposited in GeneBank^79^, 1,574,442 Trinity-assembled transcripts from 94 B73 RNA-Seq experiments^80^, 112,963 transcripts assembled from deep sequencing of a B73 seedling^81^ and 111,151 high quality transcripts from B73 Iso-seq^82^ were used as transcript evidence. In addition, the following evidences were included: 75,945 PASA transcripts from Ia453-*sh2* endosperm, 79,855 full-length transcripts from Ia453-*sh2* Iso-seq from multiple tissues, 143,679 transcripts and proteins from B73v4 annotation^18^, 36,507 transcripts and proteins from CML247 draft genome annotation^83^, 48,140 transcripts and proteins from DK105 genome annotation^21^, 46,105 transcripts and proteins from EP1 genome annotation^21^, 48,370 transcripts and proteins from F7 genome annotation^21^, 46,530 transcripts and proteins from Mo17 genome annotation^20^, 40,557 transcripts and proteins from PH207 genome annotation^84^, and 51,716 transcripts and proteins from W22 genome annotation^19^. For gene prediction, AUGUSTUS^23^ and FGENESH^24^ were trained on “maize5” and “monocots” models. The working gene set (47,168 genes and 86,182 transcripts) was identified in Ia453*-sh2* genome. All predicted proteins were annotated using InterProScan (version 5.35–74.0) and running BLASTP against UniProt database. Predicted genes were filtered according to annotation evidence distance scores (AED) calculated by MAKER-P and filtered to avoid overlapped with repeat-masked regions. In the end, 38,384 high-confidence protein-coding genes and 72,762 transcripts remained as a final set (Supplementary Fig. 3).

### TE annotation

TEs were identified in the sweet corn inbred line Ia453-*sh2* and six field corn genome assemblies (B73, Mo17, W22, EP1, F7, and DK105) using independent de novo prediction tools: EDTA v1.8.5^85^, TARGeT^86^, and SINE-Finder^87^.

#### Class I (retrotransposons)

*LTR*. LTR retrotransposons elements were predicted and annotated using EDTA package^85^. EDTA utilized a combination of LTR_FINDER^88^ and LTR harvest^89^ with LTR retriever to perform whole-genome LTR retrotransposons annotations. The redundant sequences, nested insertions and protein-coding sequences were removed by EDTA in the final non-redundant TE libraries. Intact LTR retrotransposons were identified and the insertion time of those intact LTR retrotransposons were estimated using LTR retriever^90^.

*LINE.* Long interspersed nuclear elements (LINEs) were identified using TARGeT^86^ similarity searches on the maize TE consortium (MTEC) database^77,91^, as described in detail for B73 v4^18^ genome annotation.

*SINE*. Short interspersed nuclear elements (SINEs) were annotated using SINE-Finder^87^ with default parameters (-T chunkwise -V1).

#### Class II (DNA transposons)

*TIR.* TIR-learner^92^ was used by EDTA^85^ to predict and annotate TIR transposons.

*Helitron*. HelitronScanner^93^ was used by EDTA^85^ to predict and annotate Helitron elements.

### Detection of SNPs, small indels, and structural variations

SNPs and small indels (length < X bp) were identified between Ia453-*sh2* and the other six field corn genomes using MUMmer v3.23^94^. First, nucmer from the MUMmer was used to generate the alignment with parameters “—mum –g 1000 –c 90 –l 40”. Then the alignment files were filtered to generate 1-to-1 mapping by delta-filter with parameters “-r -q”. The SNPs and small indels were called from 1-to-1 alignment blocks by show-snp with parameter “-ClrTH”. The output of NUCmer was also analyzed using Assemblytics^95^, a Web-based SV analytic tool, to identify the large structural variations (100–100,000 bp).

### OrthoMCL analysis

Orthologous gene clusters were assigned for Ia453-*sh2* and six field corn genomes using OrthoMCL^26^ with the default parameters. Splice variants and incomplete gene models in the genomes were removed, and an all-by-all comparison was then performed using BLASTP with an *E* value of 1 × 10^−5^. A total of 350,089 protein sequences were clustered into 45,600 gene families.

### Identification of Ia453-*sh2*-specific sequences, clusters, and genes

Ia453-*sh2* specific sequences were detected by first dividing the genome into sliding windows of length 1000 bp with a 500 bp overlap. Then, those small windows were aligned to the other six field corn genomes using BWA-MEM^96^ (v0.7.17) with parameters (-w 500 --M). If the sequences of windows failed to be aligned or had less than 20% of the sequences aligned to the six field corn genomes but could be perfectly aligned to the Ia453-*sh2* genome, those sequences are defined as Ia453-*sh2*-specific sequences.

The Ia453-*sh2*-specific genes were initially identified if the gene had more than 80% of CDS sequences overlapped with Ia453-*sh2* specific sequences. A second method was used to remove potential false positives. The Ia453-*sh2* CDS were aligned to the six field corn genomes using GMAP (gmap-2019-06-10) to calculate the query coverage of alignment and alignment identity^97^. The parameters query coverage of alignment and alignment identity were used to categorize the Ia453-*sh2* genes into highly conserved genes with 100% query coverage and 100% identity; genes with mutations with 100% coverage and 90-100% identity; and genes with structural variations with 50–100% coverage and 90–100% identity (Supplementary Fig. 5). The remaining genes that failed to be aligned, that had the query coverage of alignment less than 50%, or that had alignment identity less than 90% were defined as Ia453-*sh2* specific genes. The final set of Ia453-*sh2* specific genes was defined if they were detected by both methods.

Paired-end RNA-Seq reads from endosperm were aligned against Ia453-*sh2* genome using STAR v2.7.3a^98^ to check the gene expression of the identified Ia453-*sh2* specific genes. Publicly available 3′ RNAseq data from seven tissues of five sweet corn lines (P39, IL14H, Il677a, Il101T, and IA2132) and five field corn lines (B73, Mo17, W22, EP1, and F7) were used the check the gene expression of Ia453-*sh2*-specific genes in different tissues^32^.

### Structural variations of *sh2-R* allele

The *Sh2* gene sequence from B73 was aligned against Ia453-*sh2* genome using BLAST and MUMmer v3.23^94^ to compare the structural variations between *Sh2* from B73 and *sh2*-R allele in Ia453-*sh2*. Paired-end RNA-seq reads from endosperm were aligned against Ia453-*sh2* genome using STAR v2.7.3a^98^ to verify the gene expression of two predicted genes (Zm00045a021195 and Zm00045a021196) in the Ia453 *sh2-R* region.

### Gene ontology enrichment analyses

The Bioconductor package topGO (version 2.32.0) was used for the gene ontology enrichment analysis with all annotated genes as the universe set. In the topGO analysis, GO terms significance of interest were assessed based on Fisher’s exact test statistic using 0.05 as the significance threshold.

### Population genetic and phylogenetic analyses

We created a genotyping-by-sequencing build using publicly available sequences comprising 5318 diverse maize accessions were compiled from recent studies^57,99,100^. This set includes a total of 822 sweet corn line, 340 teosinte and a diverse set of tropical and temperate-adapted non-sweet lines. The TASSEL 5 GBS pipeline^101^ was used to call SNPs from all the sequencing data using the maize reference genome B73 v4, and a minimum minor allele cutoff of 10 observations, resulting in 859,632 SNPs in total. This SNP set was used for the calculation of Tajima’s *D* and XP-CLR described below. For the phylogenetic analysis, the SNPs were filtered using VCFtools^102^. SNPs were filtered to have a minor allele frequency >0.1, a linkage disequilibrium *r*^2^ < 0.2, and a percentage of missing data per SNP < 30%, resulting in a reduced set of 9725 SNPs.

A maximum likelihood-based approach was used to construct a phylogenetic tree. Maximum likelihood phylogenies were inferred with RAxML-NG^103^. Five parsimony trees and five random trees were generated to initialize the tree searches. For all ten trees, the GTR + G model was employed and an LH epsilon of 10 was chosen. The phylogenetic tree with the lowest final log likelihood at the conclusion of the tree search was chosen. The final phylogenetic tree was plotted in R with the ggplot2 and ggtree packages^104,105^. Genotype classifications were assigned and colored according to Supplementary Data 4. The same SNP set was also used to calculate principal component analysis and DAPC in the R package adegenet^106^.

### Fixation index and population structure analyses

To determine the level of genetic differentiation among different populations of maize, *F*_ST_ was calculated as described by Weir and Cockerham^107^. VCFtools was used to calculate the *F*_ST_ of every SNP between sweet corn and each other population of maize. A random set of 10,000 SNPs was used for population structure analysis. The Bayesian clustering method implemented in STRUCTURE, v.2.3.4 was used to identify clusters of genetically related individuals^108^. Ten independent replications were performed for each of *K* = 1–16 (*K* = number of genetic clusters) with a burn-in of 10,000 and 20,000 iterations. The online version of STRUCTURE HARVESTER^109^ was used to determine the optimal K based on the Evanno method^110^ (eight was the selected optimal).

### Population analysis of the *sugary1* gene

For *su1* haplotype heatmap, fasta sequences of the *sugary1* locus in 1208 genotypes were obtained by updating the B73 sequence with SNPs identified on HapMap3. A distance matrix was then calculated using the dist.dna() function from the R package phangorn^111^ with the total number of SNPs between each pair of genotypes as the distance. A corresponding heatmap of the distance matrix was then plotted using ggplot2.

For *su1* tree, a neighbor joining tree of the *su1* locus was calculated using a representative subset of 51 genotypes. The JC69 nucleotide substitution model was chosen. The tree was then rooted with the *sugary1* from *Tripsacum dactyloides* and plotted using the R packages ggplot2 and ggtree.

For *su1* multiple alignment, the *su1* locus sequence was determined from twelve overlapping PCR fragments, amplified from genomic DNA, spanning ~8.4 kb including all of the transcribed region and parts of the 5′- and 3′-UTRs. The locus was then sequenced from five introgressions of the A632 inbred background, each homozygous for an independent *su1-* mutation identified in a survey of extant sweet corn lines^33^. The *su1-nc* and *su1-sw* alleles are nearly identical to *su1-ne* (*su1-Ref)* with the exceptions of causative agent SNPs, specifically R504C in *su1-nc* and N561S in *su1-sw*, and one additional SNP in *su1-nc* compared to *su1-Ref*. In contrast, *su1-cm* and *su1-pu* were nearly identical to the B73 allele and lacked all SNPs identified in *su1-Ref*.

These results revealed two distinct *su1* haplotype groups in extant maize lines derived independently from long-divergent progenitor alleles. The group containing *su1-Ref* was designated haplotype group 1 and that containing the B73 allele as haplotype group 2. A multiple alignment of the *su1* locus was constructed on a subset of 9 genotypes including teosinte, dent corn, and the different *su1*- mutant alleles. For B73, Mo17, and PI566673, sequences were extracted from genome assemblies published at MaizeGDB. Lastly, the TIL genotypes were retrieved by updating the B73 sequence with HapMap3 SNPs.

### Identification of selection sweeps

To identify loci deviating from the mutation-drift equilibrium, we used 859,632 SNPs and calculated Tajima’s *D* for 10 kb sliding windows across the genome for the maize 282 association panel and sweet corn (along with its *sugary1* and *shrunken2* subsets). Tajima’s *D* was calculated using the R package PopGenome^112^. A locally estimated scatterplot smoothing (LOESS) was applied to the Tajima’s *D* values from each population and subsequently plotted with ggbio^113^.

To detect selective sweeps between the maize 282 association panel and sweet corn populations (along with its *sugary1* and *shrunken2* subsets), the cross-population composite likelihood ratio (XP-CLR) test was employed^114^. We used the sweet corn as a reference and the maize 282 association panel as a query to identify the selection sweeps. The selection sweeps were scanned with a sliding window of 50 kb and a step of 100 bp. The maximum number of SNPs in each window was set up as 50 and the correlation levels were set up as 0.7. XP-CLR values from the three comparisons were then plotted with ggbio^113^. The regions with XP-CLR values in the top 1% of the empirical distributions were designated as candidate sweeps and the candidate genes were identified within the selected candidate genomic regions. We note that other genes within the selected candidate regions may actually be the ones originally associated with the selective sweep, and future research will be required to validate and establish the functional link between the causal gene and the sweep.

### Reporting summary

Further information on research design is available in the Nature Research Reporting Summary linked to this article.

## Supplementary information

Supplementary Information

Reporting Summary

Description of Additional Supplementary Files

Supplementary Data 1

Supplementary Data 2

Supplementary Data 3

Supplementary Data 4

## Data Availability

Data supporting the findings of this work are available within the paper and its Supplementary Information files. A reporting summary for this Article is available as a Supplementary Information file. All other raw data are available from the corresponding author upon request. The genome assembly have been deposited in NCBI database under BioProject accession PRJNA646414, BioSample accession SAMN15543012, and GeneBank accession JACHTI000000000.1/. RNA-seq data have been deposited in NCBI Sequence Read Archive under accession SRR12300193, SRR12300194, SRR12300195, and SRR12300196, as well as in BioProject under accession PRJNA647770. The protein coding gene, transposon annotations, and the VCF file of 5381 maize lines are publicly available at CyVerse. Source data are provided with this paper.

## Source data

Source Data
