# Supplementary material for: Genome assembly and population genomic analysis provide insights into the evolution of modern sweet corn
Source: Nat Commun. 2021 Feb 23;12:1227. doi: 10.1038/s41467-021-21380-4 (PMC7902669; doi:10.1038/s41467-021-21380-4)
Supplement: Supplementary file 2 — Reporting Summary [file 41467_2021_21380_MOESM2_ESM.pdf]

nature research

Corresponding author(s): Marcio F. R. Resende

Last updated by author(s): Dec 26, 2020

Reporting Summary

Nature Research wishes to improve the reproducibility of the work that we publish. This form provides structure for consistency and transparency in reporting. For further information on Nature Research policies, see our Editorial Policies and the Editorial Policy Checklist.

Statistics

For all statistical analyses, confirm that the following items are present in the figure legend, table legend, main text, or Methods section.

n/a	Confirmed	
☐	☒	The exact sample size (*n*) for each experimental group/condition, given as a discrete number and unit of measurement
☐	☒	A statement on whether measurements were taken from distinct samples or whether the same sample was measured repeatedly
☒	☐	The statistical test(s) used AND whether they are one- or two-sided<br>*Only common tests should be described solely by name; describe more complex techniques in the Methods section.*
☒	☐	A description of all covariates tested
☒	☐	A description of any assumptions or corrections, such as tests of normality and adjustment for multiple comparisons
☐	☒	A full description of the statistical parameters including central tendency (e.g. means) or other basic estimates (e.g. regression coefficient) AND variation (e.g. standard deviation) or associated estimates of uncertainty (e.g. confidence intervals)
☐	☒	For null hypothesis testing, the test statistic (e.g. F, t, r) with confidence intervals, effect sizes, degrees of freedom and P value noted<br>*Give P values as exact values whenever suitable.*
☐	☒	For Bayesian analysis, information on the choice of priors and Markov chain Monte Carlo settings
☒	☐	For hierarchical and complex designs, identification of the appropriate level for tests and full reporting of outcomes
☒	☐	Estimates of effect sizes (e.g. Cohen's *d*, Pearson's *r*), indicating how they were calculated

Our web collection on statistics for biologists contains articles on many of the points above.

Software and code

Policy information about availability of computer code

Data collection	No software was used in data collection.
Data analysis	Canu version 1.8; BioNano Solve 3.2 software; BioNano Access 1.4.3 software; minimus2 version 3.1.0; A python-based command line tool reform (https://github.com/gencorefacility/reform) was used to put back the merged sequences into the hybrid assembly; SNAP (http://snap.cs.berkeley.edu/); ntedit version 1.2.1; jellyfish version 2.3.0; BUSCO version 3.02; ltrretriever version 2.5; IsoSeq3 version 3.1 (https://github.com/PacificBiosciences/IsoSeq3); Trimmomatic version 0.36; PEAR version 0.9.6; Trinity version 2.8.4; HISAT2 version 2.1.0; StringTie version 1.3.4; PASA version 2.2.0; MAKER-P version 2.31.10; InterProScan version 5.35-74.0; EDTA v1.8.5; TARGeT (http://target.iplantcollaborative.org/); SINE-Finder (http://www.plantcell.org/content/suppl/2011/08/29/tpc.111.088682.DC1); LTR harvest version 1.5.9; LTR finder version 1.0.5; MUMmer version 3.23; Assemblytics (http://assemblytics.com/); OrthoMCL v.1.4; BWA-MEM version 0.7.17; gmap version 2019-06-10; STAR version 2.7.3a; TASSEL 5 GBS pipeline; VCFtools version 0.1.16; raxml-ng version 0.9.0; R with the ggplot2, ggtree, phangorn, PopGenome, ggbio, topGO and adegenet packages; STRUCTURE version 2.3.4; STRUCTURE HARVESTER (http://taylor0.biology.ucla.edu/structureHarvester/); xpclr version 1.1.1; BD Accuri CFlow software (Version 1.0.264; BD Biosciences, CA, USA)

For manuscripts utilizing custom algorithms or software that are central to the research but not yet described in published literature, software must be made available to editors and reviewers. We strongly encourage code deposition in a community repository (e.g. GitHub). See the Nature Research guidelines for submitting code & software for further information.

Data

Policy information about availability of data

All manuscripts must include a data availability statement. This statement should provide the following information, where applicable:

- Accession codes, unique identifiers, or web links for publicly available datasets
- A list of figures that have associated raw data
- A description of any restrictions on data availability

The genome assembly have been deposited in NCBI database under BioProject number PRJNA646414, BioSample number SAMN15543012 and GeneBank accession number JACHTI000000000. RNA-seq data were deposited in the National Center of Biotechnology Information Sequence Read Archive under accession number SRR12300193, SRR12300194, SRR12300195 and SRR12300196 and in BioProject under accession number PRJNA647770. Protein-coding gene annotation, transposon annotation, and vcf file of 5,381 maize accessions are available on Cyverse (/iplant/home/shared/commons_repo/curated/UF_resende_Sweet_corn_2020). Raw sequence data are available upon request. Databases used in manuscript include AUGUSTUS database “maize5”, FGENESH database "monocots", Plantae BUSCO ‘Embryophyta_odb9’ database, UniProt database, and maize TE consortium (MTEC) database.

Field-specific reporting

Please select the one below that is the best fit for your research. If you are not sure, read the appropriate sections before making your selection.

☐ Life sciences ☐ Behavioural & social sciences ☒ Ecological, evolutionary & environmental sciences

For a reference copy of the document with all sections, see nature.com/documents/nr-reporting-summary-flat.pdf

Ecological, evolutionary & environmental sciences study design

All studies must disclose on these points even when the disclosure is negative.

Study description	We sequenced and de novo assembled a sweet corn inbred line, Ia453, with the mutated shrunken2-Reference allele (Ia453-sh2). This mutation accumulates more sugar and is present in most commercial hybrids developed for the processing and fresh markets. The ten pseudochromosomes covered 92% of the total assembly and 99% of the estimated genome size, with an N50 of 222.2Mb. This reference genome completely assembled the large structural variation that created the mutant sh2-R allele. Furthermore, comparative genomics with six field corn genomes highlights differences in single-nucleotide polymorphisms, structural variations and transposon composition. Phylogenetic analysis of 5,318 diverse maize and teosinte accessions revealed genetic relationships between modern sweet corn and other types of maize. Our results show evidence for a common origin in northern Mexico for modern sweet corn in the U.S. Finally, population genomic analysis identified regions of the genome under selection and candidate genes associated with sweet corn traits, such as early flowering, endosperm composition, plant and tassel architecture, and kernel row number.
Research sample	Sweet corn (Zea mays L.) is grown all over the world and is one of the most important vegetables in the United States and Canada. Ia453-sh2 is an important public inbred line which contributed to the establishment of sh2 sweet corn. The sweet corn (Zea mays L.) inbred line Ia453 with sh2-R allele (Ia453-sh2) used for PacBio sequencing were grown in the greenhouse complex at the University of Florida (Gainesville, FL) in November of 2018. Young leaves from 3-week-old plants were harvested and frozen in liquid nitrogen. RNA-seq data was also generated from endosperm sampled 14 days after pollination. Iso-seq data was also generated from leaf, stem, silk, husk, ear and pollen tissues. A new Genotyping-By-Sequencing build was created using publicly available sequences comprising 5,318 diverse maize accessions compiled from recent studies including teosinte, landraces, field corns and sweet corns. More details are in the 1st paragraph of M&M.
Sampling strategy	For the primary experiments the sample size was 1 (for the genome assembly and annotation). DNA from 1-week old etiolated seedings was extracted for PacBio sequencing. PacBio iso-seq sampled RNA from a broad group of plant tissues. Population genetic analysis used all samples available. No sample size calculation was required for this study.
Data collection	Large insert (20 kb) SMRTbell libraries were prepared and sequenced using a PacBio SEQUEL system; All Illumina libraries were prepared and sequenced with 150 bp paired-end reads on an Illumina Hiseq 2500 system; The BioNano Saphyr system was used to stretch, separate and image the labeled DNA molecules; A Hi-C library (Dovetail Genomics LLC, Santa Cruz, CA) was generated using the DpnII restriction endonuclease (GATC) and the Dovetail Hi-C library were used to perform scaffolding through Dovetail’s HiRise pipepline.
Timing and spatial scale	Pacbio data collections started on January 19 2018 and ended on March 8 2018 by the Interdisciplinary Center for Biotechnology Research (ICBR) at the University of Florida. Illumina data collection started on January 16 2018 and ended on February 6 2018 by at GENEWIZ Inc. (South Plainfield, NJ). Bionano data collection started on April 18 2019 and ended at June 6 2019 by Bionano Genomics (Johnston, IA). Dovetail Hi-C data collection started on May 18 2018 and ended on July 17 2019 by Dovetail Genomics LLC (Santa Cruz, CA). As the sequencing data was collected at fully automated machines, the data collection was finished at one-shot.
Data exclusions	There is no data excluded from the analyses.
Reproducibility	Most experiments were not replicated as they report the results of genome assembly and annotation. For the RNA-Seq experiment, we used three biological replicates.
Randomization	Not relevant to the study since a single inbred line was sequenced.

Blinding	The investigators are blinded to group allocation during data collection. The investigators were blinded to the outcomes of clustering analysis.

Did the study involve field work? ☐ Yes ☒ No

Reporting for specific materials, systems and methods

We require information from authors about some types of materials, experimental systems and methods used in many studies. Here, indicate whether each material, system or method listed is relevant to your study. If you are not sure if a list item applies to your research, read the appropriate section before selecting a response.

Materials & experimental systems

n/a	Involved in the study
☒	☐ Antibodies
☒	☐ Eukaryotic cell lines
☒	☐ Palaeontology and archaeology
☒	☐ Animals and other organisms
☒	☐ Human research participants
☒	☐ Clinical data
☒	☐ Dual use research of concern

Methods

n/a	Involved in the study
☒	☐ ChIP-seq
☐	☒ Flow cytometry
☒	☐ MRI-based neuroimaging

Flow Cytometry

Plots

Confirm that:

☒ The axis labels state the marker and fluorochrome used (e.g. CD4-FITC).

☒ The axis scales are clearly visible. Include numbers along axes only for bottom left plot of group (a 'group' is an analysis of identical markers).

☐ All plots are contour plots with outliers or pseudocolor plots.

☐ A numerical value for number of cells or percentage (with statistics) is provided.

Methodology

Sample preparation	Ia453-sh2, F7, W22 and B73 were used to estimate genome size using flow cytometry. Flow cytometry measurements were made on five biological replicates per genotype, and the estimated genome size was reported as the average across the five replicates. Five seeds per genotype were imbibed for 48 hours in full darkness, and the embryo was dissected and mixed with 500 µl of extraction buffer.
Instrument	BD Accuri C6 Flow Cytometer using the FL2 channel (Accuri Cytometers, Ann Arbor, MI)
Software	BD Accuri CFlow software
Cell population abundance	For each sample at least 5,000 nuclei were counted and analyzed using the BD Accuri CFlow software. In this ploidy analysis cell sorting and purification steps were not involved.
Gating strategy	The gating strategy is provided in the Supplementary Figure S1. B73 was used as the diploid template.

☒ Tick this box to confirm that a figure exemplifying the gating strategy is provided in the Supplementary Information.
