# Supplementary material for: Genome assembly and population genomic analysis provide insights into the evolution of modern sweet corn
Source: Nat Commun. 2021 Feb 23;12:1227. doi: 10.1038/s41467-021-21380-4 (PMC7902669; doi:10.1038/s41467-021-21380-4)
Supplement: Supplementary file 3 — Description of Additional Supplementary Files [file 41467_2021_21380_MOESM3_ESM.pdf]

Description of Additional Supplementary Files

Supplementary Data 1. Summary of TE superfamily in Ia453-sh2 and six field corn genomes

Supplementary Data 2. Structural variations between Ia453-sh2 and six field corn genomes

Supplementary Data 3. Significant enriched GO terms of core genes shared by Ia453-sh2 and six field corn genomes. GO term enrichment was tested using Bioconductor package topGO. GO terms significance of interest were assessed based on Fisher's exact test statistic using 0.05 as the significance threshold.

Supplementary Data 4. Genotype classifications used to color the phylogenetic tree presented in Figure 3. GroupID was used to define the groups. GroupID 'Unclassified' was colored light green (#8DD3C7) and was used for unknown genotypes.
