# Supplementary material for: Genome assembly and population genomic analysis provide insights into the evolution of modern sweet corn
Source: Nat Commun. 2021 Feb 23;12:1227. doi: 10.1038/s41467-021-21380-4 (PMC7902669; doi:10.1038/s41467-021-21380-4)
Supplement: Supplementary file 1 — Supplementary Information [file 41467_2021_21380_MOESM1_ESM.pdf]

Genome assembly and population genomic analysis provide insights into the evolution of modern sweet corn

Hu *et al.*

Supplementary Table 1. Number and characteristics of contigs and scaffolds for each of the Ia453-*sh2* assemblies

	PacBio assembly	BioNano assembly	Dovetail assembly	Pseudomolecule
Total length of assembly (Gb)	2.25	2.32	2.29	2.11
Number of contigs (scaffolds)	15,550	8,515	8,450	10
N50 (Mb)	0.39	120.95	222.20	222.20
Maximum length (Mb)	2.58	255.03	304.49	304.49

Supplementary Table 2. Flow cytometry analysis and genome size estimated as a ratio relative to maize B73 genome size.

Genotype	Average G1	Standard Error	Ratio	n
B73	247544.7	2206.1	1.00	5
Ia453-*sh2*	260437.6	2309.5	1.05	5
F7	255884.0	1736.4	1.03	5
W22	263452.0	1195.0	1.06	5

Supplementary Table 3. Comparisons of the completeness of Ia453-*sh2* and six field corn genomes by BUSCOs

Gene type	Ia453-*sh2*	B73v4	W22	Mo17	F7	EP1	DK105
Complete single-copy BUSCOs	1268 (88.05%)	1277 (88.68%)	1281 (88.95%)	1262 (87.64%)	1273 (88.40%)	1287 (89.37%)	1286 (89.31%)
Complete duplicated BUSCOs	95 (6.59%)	89 (6.18%)	85 (5.90%)	99 (6.87%)	90 (6.25%)	85 (5.90%)	82 (5.69%)
Fragmented BUSCOs	18 (1.11%)	16 (1.11%)	23 (1.59%)	25 (1.74%)	26 (1.81%)	19 (1.32%)	28 (1.94%)
Missing BUSCOs	59 (4.09%)	58 (4.02%)	51 (3.54%)	54 (3.75%)	51 (3.54%)	49 (3.40%)	44 (3.05%)

Supplementary Table 4. Insertion time and family of intact LTRs in *sh2-R* allele

LTR	Start	End	Length	Super family	TE type	Insertion time
Intact LTR 1	223,920,452	223,929,925	9473	Copia	LTR	293,799
Intact LTR 2	223,941,131	223,952,889	11758	Gypsy	LTR	578,846
Intact LTR 3	223,959,794	223,965,239	5445	Copia	LTR	0

Supplementary Table 5. Summary of one-to-one syntenic sequence, SNPs, small indels and Ia453-*sh2* specific sequence in pairwise comparisons between Ia453-*sh2* and six field corn genomes

**Ia453-*sh2* compared to six field corn genomes**		**B73v4**	**Mo17**	**W22**	**DK105**	**EP1**	**F7**
**One-to-one syntenic blocks**	Length (bp)	1,562,161,471	1,556,250,045	1,564,739,926	1,611,235,583	1,598,318,073	1,598,547,486
	Percentage of genome (%)	68.09%	67.83%	68.21%	70.23%	69.67%	69.68%
**SNPs**	# SNPs	10,694,465	10,725,464	10,694,960	9,316,470	10,935,730	10,592,122
	Percentage of genome (%)	0.466%	0.468%	0.466%	0.406%	0.477%	0.462%
**Small insertions (<100bp)**	# small insertions	455,957	484,014	448,971	403,845	463,046	443,359
	Length (bp)	2,579,125	2,576,690	2,513,714	2,265,686	2,583,973	2,473,313
	Percentage of genome (%)	0.112%	0.112%	0.109%	0.099%	0.113%	0.108%
**Small deletions (<100bp)**	# small deletions	367,123	365,674	358,251	321,422	370,378	355,606
	Length (bp)	2,455,461	2,429,165	2,366,345	2,172,091	2,482,738	2,372,479
	Percentage of genome (%)	0.107%	0.106%	0.103%	0.095%	0.108%	0.103%
**Ia453-*sh2* specific regions**	Length (bp)	61,855,159	61,426,531	64,306,208	56,665,231	61,498,986	60,453,017
	Percentage of genome (%)	2.696%	2.678%	2.803%	2.470%	2.681%	2.635%

Supplementary Table 6. Summary of six Ia453-*sh2* specific genes

Gene ID	Chromosome	Start	End	Description
Zm00045a011525	Chr2	230350730	230354560	Similar to ALDH6A1 Methylmalonate-semialdehyde dehydrogenase [acylating], mitochondrial (Homo sapiens OX=9606)
Zm00045a021614	Chr3	237420369	237422288	Similar to At4g03220 Putative F-box/FBD/LRR-repeat protein At4g03220 (Arabidopsis thaliana OX=3702)
Zm00045a028561	Chr8	119646727	119647100	Protein of unknown function
Zm00045a030178	Chr8	176620727	176625519	Similar to SPHK2 Sphingosine kinase 2 (Arabidopsis thaliana OX=3702)
Zm00045a030528	Chr6	9812667	9813181	Protein of unknown function
Zm00045a046064	Scaffold_829	15858	16661	Similar to PAE1 Proteasome subunit alpha type-5 (Glycine max OX=3847)

Supplementary Table 7. Comparison of the input assembly and the final HiRise scaffolds.

	Input assembly	Dovetail HiRise assembly
Total length	2,293.85 Mb	2,293.85 Mb
L50/N50	7 scaffolds/119.59Mb	5 scaffolds/222.196Mb
L90/N90	21 scaffolds/21.444Mb	10 scaffolds/150.288Mb
Longest scaffold	251,409,103 bp	304,485,272 bp
Number of scaffolds	8,689	8,440
Number of scaffolds > 1kb	8,648	8,573
Number of gaps	11,661	11,686
Percent of genome in gaps	2.65%	2.65%

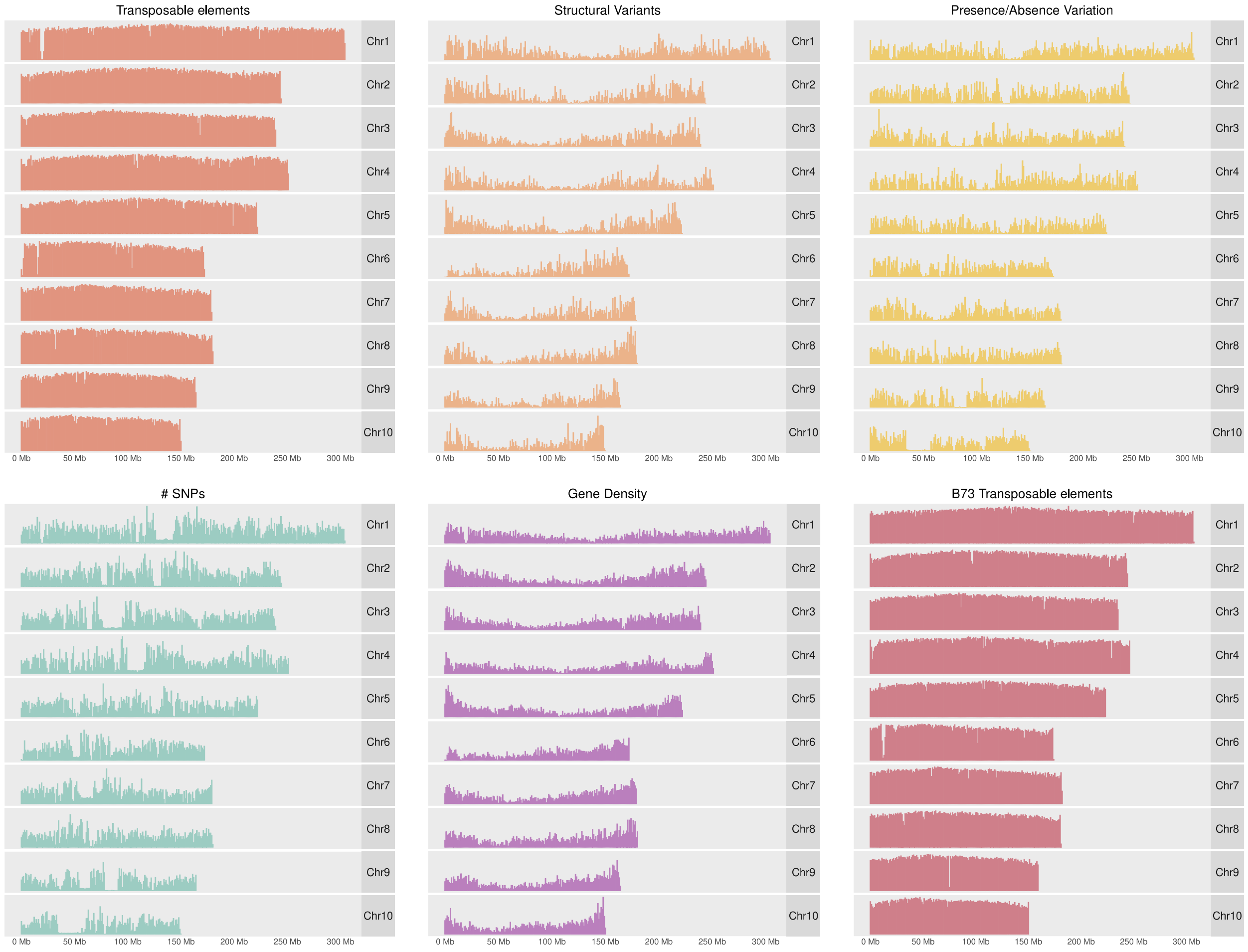

Supplementary Figure 1. Linear representation of Ia453-*sh2* genomic features including transposable-elements (TEs) (fraction of TEs per 1 Mb window); large structural variations (SVs) with length between 100 and 100,000 bps (fraction of SVs per 1 Mb window) relative to B73 v4; presence and absence variants (PAV) (fraction of PAVs per 1 Mb window) relative to B73 v4; Single nucleotide polymorphisms (SNPs) (number of SNPs per 1 Mb window) relative to B73 v4; gene density (number of genes per 1 Mb window); and transposable-elements (TEs) (fraction of TEs per 1 Mb window) of B73 v4 for comparison (bottom right).

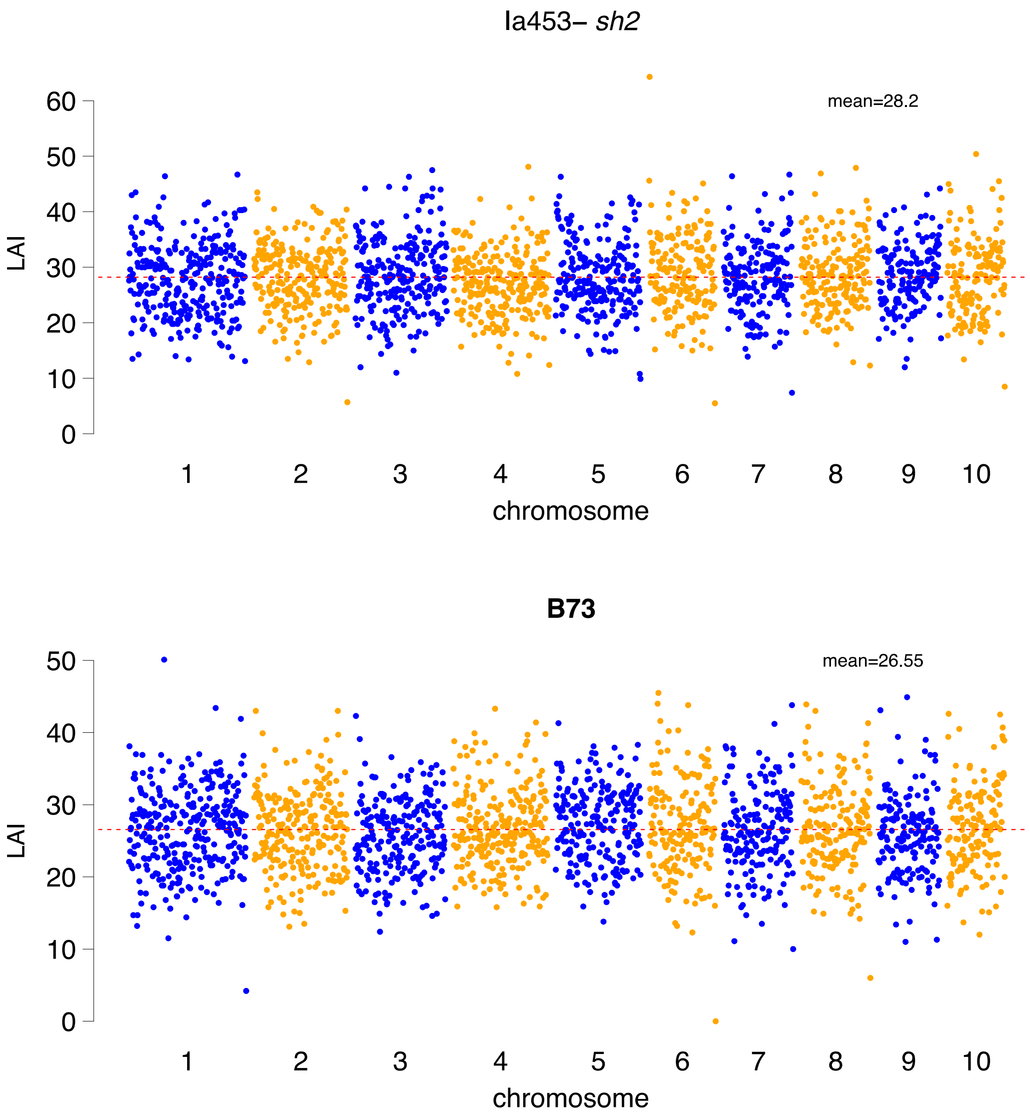

Supplementary Figure 2. LAI scores in 10 pseudochromosomes of Ia453-*sh2* and B73 v4 genomes. Each dot represents LAI score with 1-Mb sliding window.

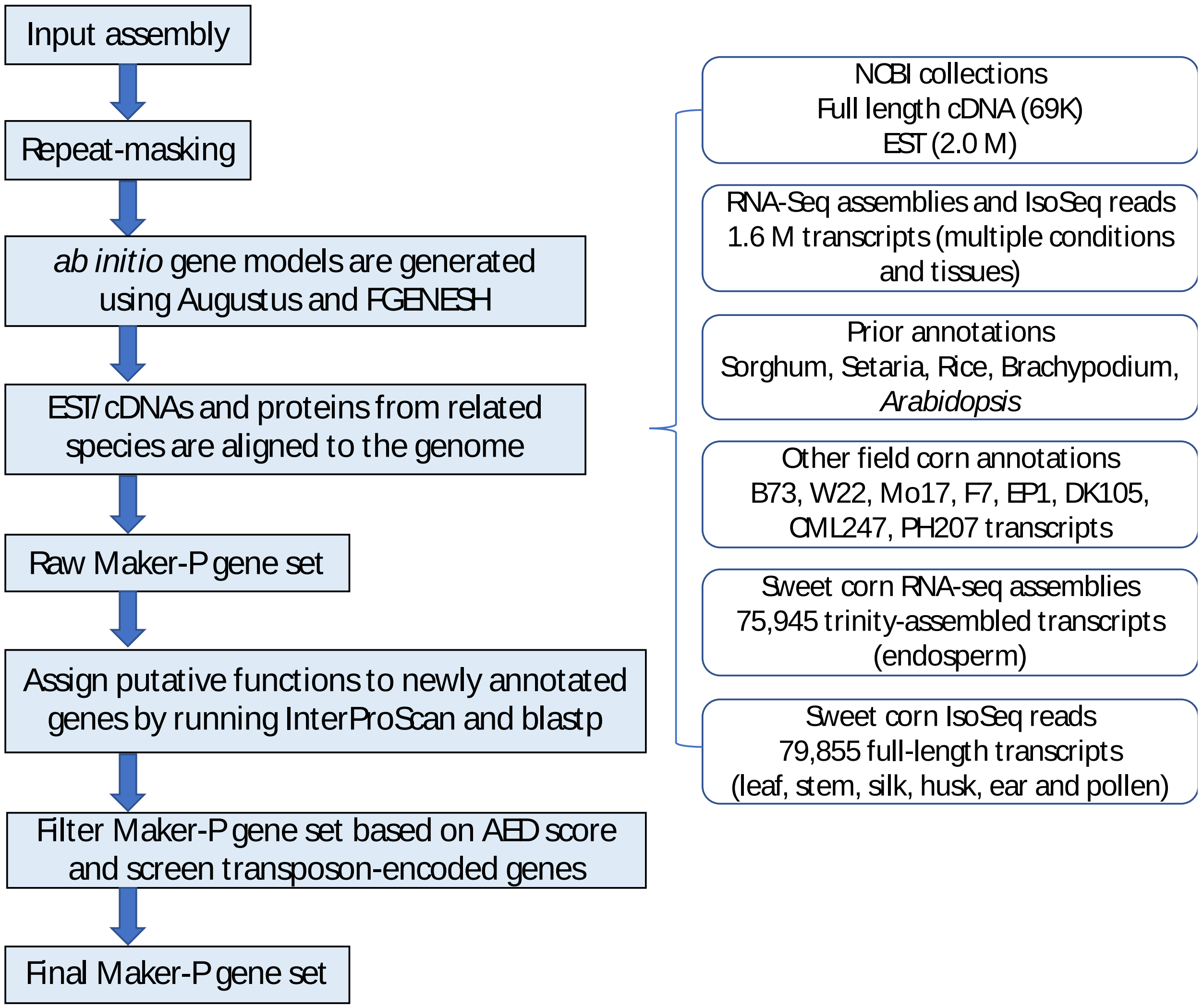

Supplementary Figure 3. Workflow for protein coding gene annotation. Multiple evidences shown in the rectangles on the right were used to annotate genes in the Ia453-*sh2* genome.

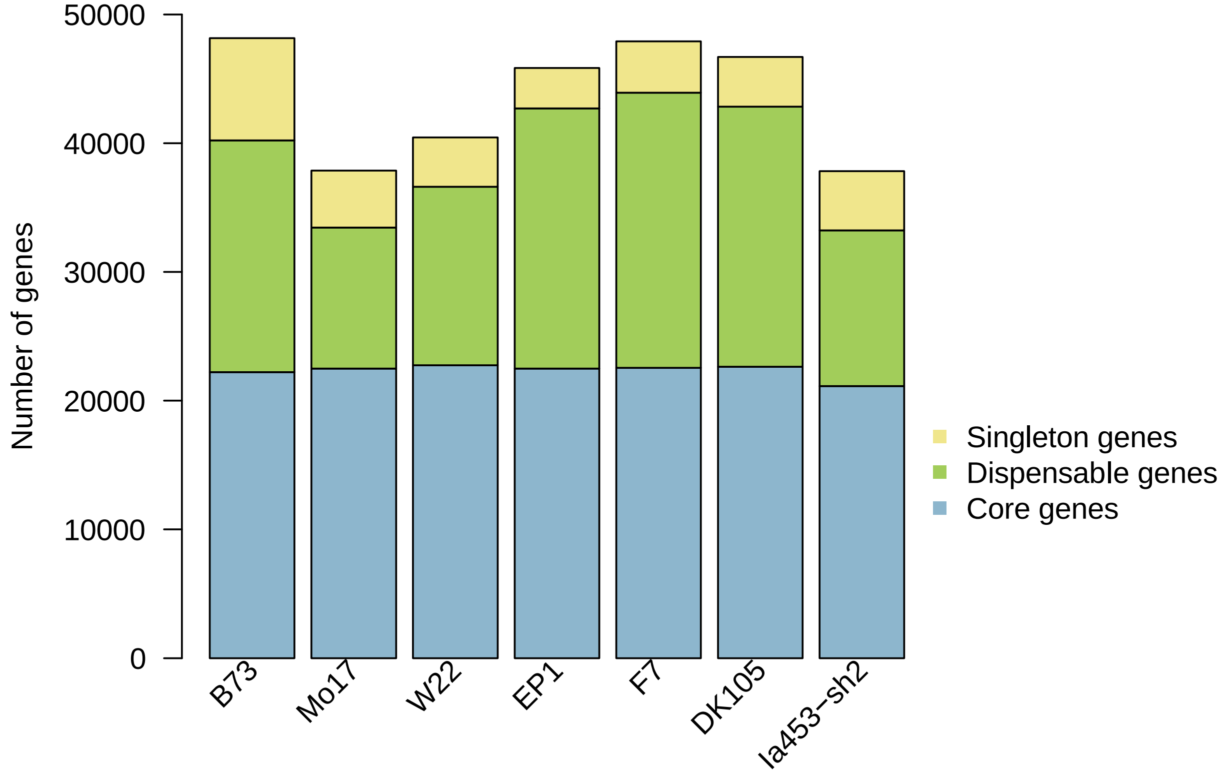

Supplementary Figure 4. Number of core, dispensable and unique genes in Ia453-*sh2* and other six field corn genomes. Source data are provided as a Source Data file.

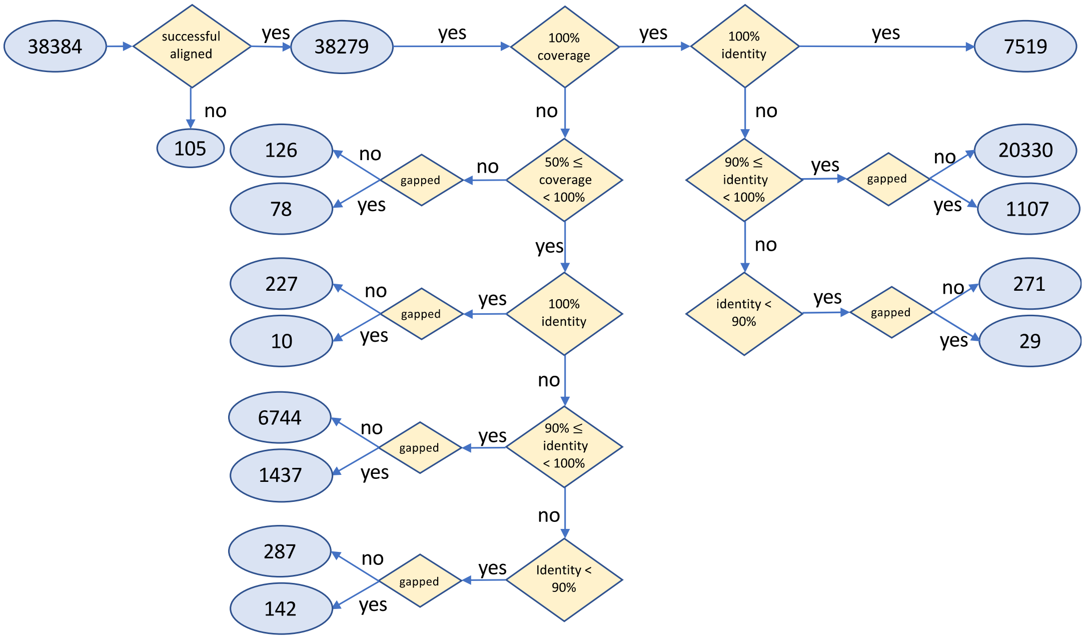

Supplementary Figure 5. Workflow for identifying Ia453-*sh2* specific genes compared to B73. A total of 38,384 protein coding genes in Ia453-*sh2* was aligned to B73 v4 genome. There are 38,279 genes which can be successfully aligned to B73 v4 genome while 105 genes cannot be aligned. Out of the aligned genes, there are 7,519 highly conserved genes between Ia453-*sh2* and B73 v4. With 100% query coverage, there are 21,437 genes with 90-100% identity and 300 genes with 100% query coverage and less than 90% identity. With query coverage 50-100%, there are 237 genes with 100% identity, 8,181 genes with 90-100% identity and 429 genes with less than 90% identity. There are 204 genes have less than 50% query coverage.

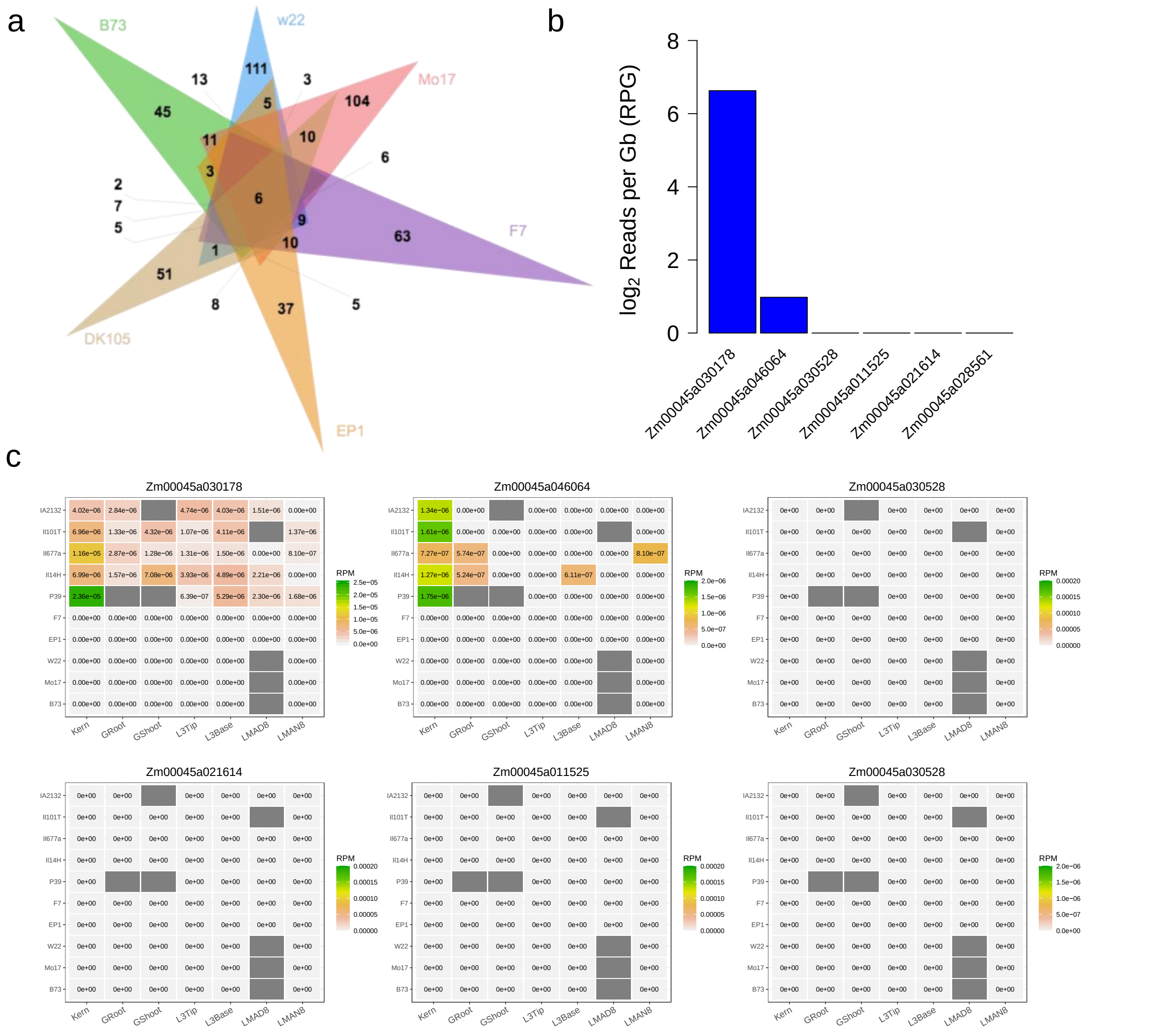

Supplementary Figure 6. Identification of Ia453-*sh2* specific genes. a, Venn diagram of the Ia453-*sh2* specific genes along the six comparisons with six field corn genomes. b, Log_2 transformed reads per Gb (RPG) of the six Ia453-*sh2* specific genes. c, Heatmap showing the expression of the six Ia453-*sh2* specific genes in different tissues (Kern: Kernel; GRoot: Germinating root; GShoot: Germinating shoot; L3Tip: L3Base; LMAN8:) of 5 sweet corn lines (P39, Il14H, Il677a, Il101T, and IA2132) and 5 field corn lines (B73, Mo17, W22, EP1 and F7) by using publicly available 3' RNAseq data. Source data underlying Supplementary Figure 6b are provided as a Source Data file.

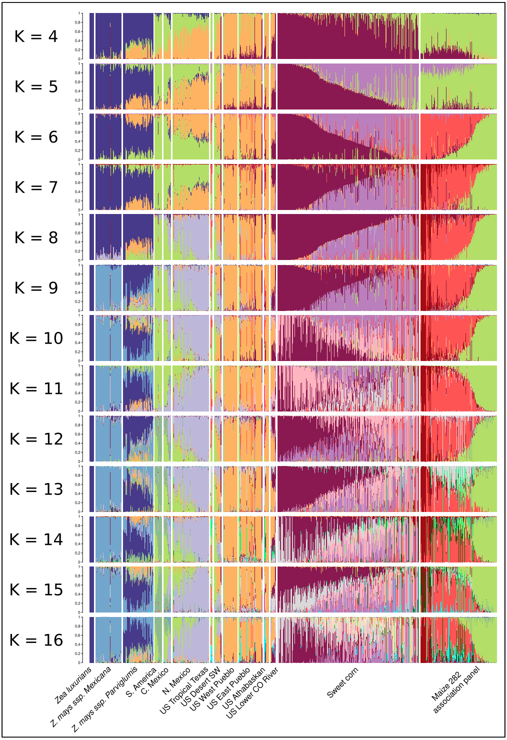

Supplementary Figure 7. Bayesian clustering of the population using STRUCTURE with K = 4-16.

11

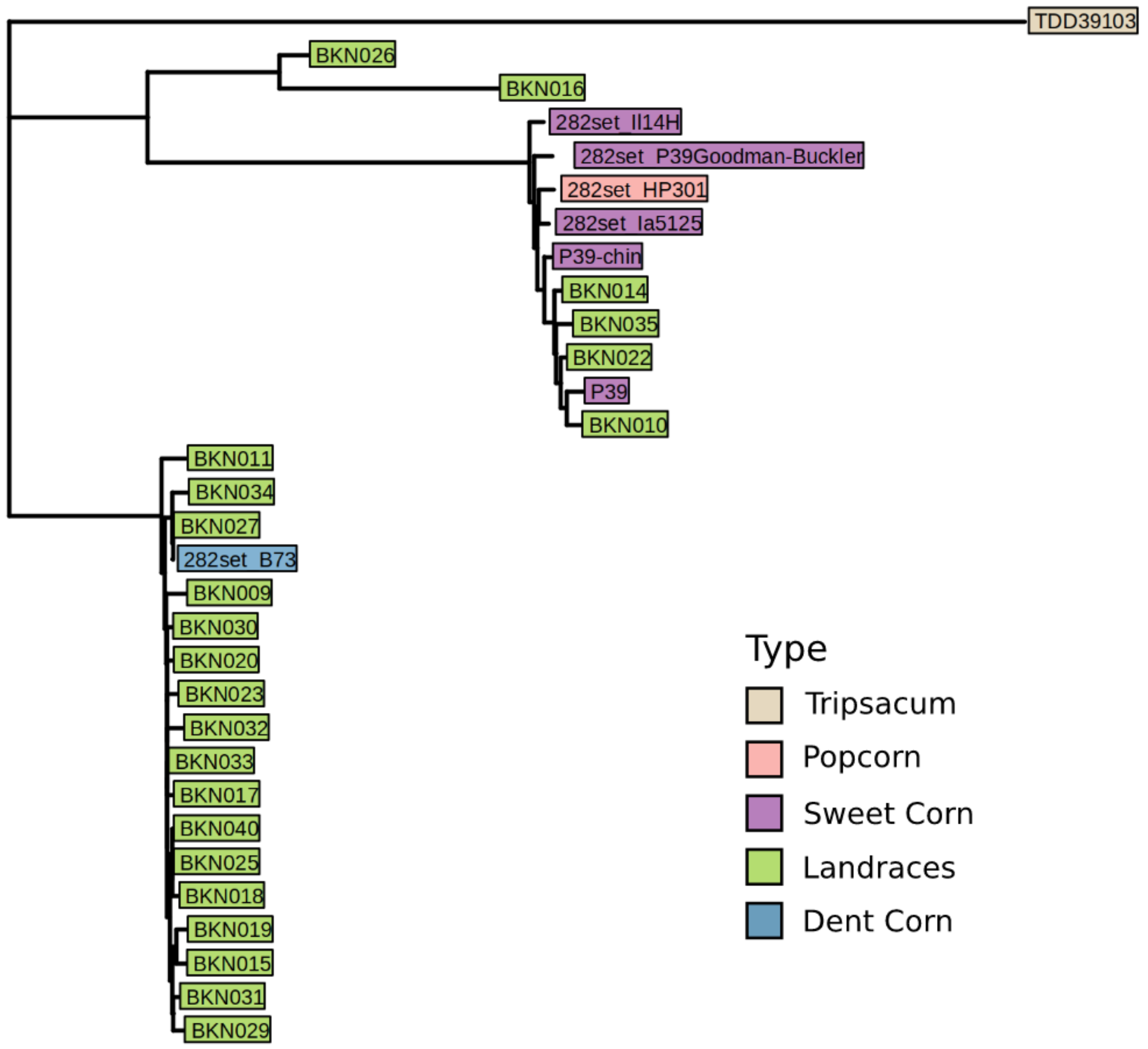

Supplementary Figure 8. Neighbor-joining *su1* tree rooted in *Tripsacum dactyloides*, containing landraces and representatives of the two dominant *su1* haplotypes.

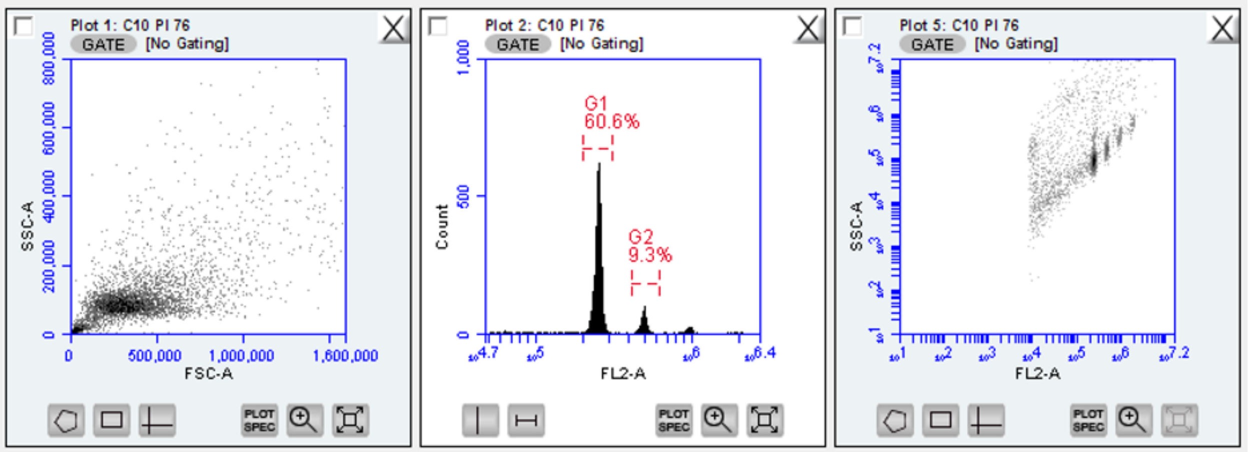

Plot 2: C10 PI 76	Count	Volume (µL)	% of This Plot	% of All	Mean FL2-A	CV FL2-A	Median FL2-A
All	5,030	134	100.00%	100.00%	303,332.77	116.66%	
G1 (198,653.0 / 3…	3,048	134	60.60%	60.60%	251,976.20	5.48%	
G2 (410,767.0 / 6…	467	134	9.28%	9.28%	498,158.72	5.77%	

Supplementary Figure 9. Flow cytometry gating strategy. G1 and G2 peaks were manually gated in the histogram (center) to record the mean FL2-A and coefficient of variation (CV) for FL2-A for each peak. The positions of the G1 peaks were averaged for five biological replicates for each genotype.
